# Causal Artificial Intelligence in Legal Language Processing: A Systematic Review

**DOI:** 10.3390/e27040351

**Published:** 2025-03-28

**Authors:** Philippe Prince Tritto, Hiram Ponce

**Affiliations:** 1 Facultad de Derecho, Universidad Panamericana, Augusto Rodin 498, Mexico City 03920, Mexico; 2 Facultad de Ingeniería, Universidad Panamericana, Augusto Rodin 498, Mexico City 03920, Mexico; hponce@up.edu.mx

**Keywords:** causal artificial intelligence, causal machine learning, legal language processing, legal AI, natural language processing, legal reasoning, systematic review, causal inference, machine learning, legal text analysis

## Abstract

Recent advances in legal language processing have highlighted limitations in correlation-based artificial intelligence approaches, prompting exploration of Causal Artificial Intelligence (AI) techniques for improved legal reasoning. This systematic review examines the challenges, limitations, and potential impact of Causal AI in legal language processing compared to traditional correlation-based methods. Following the Joanna Briggs Institute methodology, we analyzed 47 papers from 2017 to 2024 across academic databases, private sector publications, and policy documents, evaluating their contributions through a rigorous scoring framework assessing Causal AI implementation, legal relevance, interpretation capabilities, and methodological quality. Our findings reveal that while Causal AI frameworks demonstrate superior capability in capturing legal reasoning compared to correlation-based methods, significant challenges remain in handling legal uncertainty, computational scalability, and potential algorithmic bias. The scarcity of comprehensive real-world implementations and overemphasis on transformer architectures without causal reasoning capabilities represent critical gaps in current research. Future development requires balanced integration of AI innovation with law’s narrative functions, particularly focusing on scalable architectures for maintaining causal coherence while preserving interpretability in legal analysis.

## 1. Introduction

Legal language processing is an application of artificial intelligence (AI) techniques for natural language processing (NLP) to legal language. NLP is a major area of research and development in the field of information technology, attempting to replicate human abilities to process text. The ultimate purpose of NLP is natural language understanding (NLU). According to Elizabeth Liddy [1], a full NLU system would be able to paraphrase text, translate it, answer questions about its contents, and draw inferences from it.

It is now accepted that the goal of NLP is the true understanding that can be expected from a machine. Answering specific questions according to input text is one of the objectives that particularly interests us in our research, since legal problems may arise when deciding. Decisions in NLP may have subtle legal overtones, and the decisions that the machine must make when dealing automatically with natural language may face legal dilemmas, such as ensuring that the inferences generated from a text do not present a racist bias despite allowing a more efficient delivery of justice.

### 1.1. NLP and the Open Texture of Legal Language

In the field of NLP, technical language is easier to interpret than ordinary language, due to the low polyvalence of the meaning of its words. However, law represents a unique challenge, as illustrated by Hart’s analysis of a simple rule “prohibiting the use of vehicles in the park” [2]. While this rule clearly applies to cars, Hart demonstrates how edge cases like bicycles, roller skates, or toy cars create interpretative challenges. This exemplifies what Hart calls the *open texture* of law: “uncertainty at the borderline is the price to be paid for the use of general classifying terms in any form of communication concerning matters of fact” [2].

The interpretation of legal language is thus central to legal praxis, since causal relationships between verbal elements and human experience condition the understanding of the text and the adoption of solutions.

### 1.2. Causality in Theoretical Computer Science and Its Use in NLP

In computer science, causality provides a fundamental framework for understanding and modeling how systems produce their effects. While traditional AI approaches focus on identifying statistical patterns in data, Causal AI (while some literature distinguishes between Causal Machine Learning (focusing specifically on machine learning techniques) and Causal AI (encompassing broader artificial intelligence approaches), we use Causal AI as an umbrella term that includes machine learning applications, consistent with our systematic search methodology detailed in Section 2) represents a paradigm shift toward systems that can reason about cause-and-effect relationships. This shift is particularly significant in natural language processing, where moving beyond statistical correlations (we use the word “association” to refer to any general relationship between two variables, indicating that certain values of one variable tend to co-occur with certain values of another. This term is broad and encompasses all types of relationships, whether they are linear or non-linear, and applies to various data types, including nominal, ordinal, interval, and ratio scales [3]. We use the word “correlation” as a specific statistical measure, a type of association that quantifies the strength and direction of a linear relationship between two quantitative variables [3])to causal understanding is crucial for improving the robustness, fairness, and interpretability of language models [4]. These systems combine computational methods with formal theories of causation to model not just what happens, but why and how it happens.

The foundations of modern causal inference trace back to the Neyman–Rubin causal model, which formalizes causation through the concept of potential outcomes [5]. This framework defines causal effects as the difference between potential outcomes under treatment and control conditions, though only one outcome can ever be observed for any unit [6]. While originally developed for experimental settings, this model has profound implications for observational studies in law, where researchers must carefully consider counterfactuals and selection bias [7]. The framework’s emphasis on explicit counterfactual reasoning aligns naturally with legal analysis, where courts frequently engage in counterfactual inquiry—considering what would have happened “but for” a defendant’s actions [8]. Modern Causal AI approaches in legal language processing build upon these foundations while addressing the unique challenges of extracting causal relationships from unstructured text [4].

Causal AI systems are distinguished by three key capabilities [4,9,10]: first, they can answer “what if” questions through interventional reasoning, allowing them to predict the effects of actions even in previously unseen scenarios. Second, they can evaluate counterfactuals, determining what would have happened under different circumstances. Third, they explicitly model the mechanisms that generate data, rather than merely capturing statistical correlations. In the context of language processing, these capabilities extend to understanding how textual interventions affect outcomes, evaluating counterfactual text variations, and modeling the underlying generative processes of language [4].

The field encompasses a vast array of methodologies, from classical approaches like Regression Discontinuity Designs and Difference-in-Differences to modern techniques like Dynamic Treatment Regimes and Synthetic Controls, though not all have yet been applied to legal NLP.

Structural Causal Models (SCMs) provide a mathematical foundation for representing causal relationships [11]. Causal diagrams model the world based on prior knowledge, making explicit the assumptions about how variables influence each other [10]. In the context of natural language processing, unique challenges emerge when applying these frameworks to text data [4], including the high dimensionality of language representations, the need for sophisticated modeling to measure semantically meaningful factors, and careful consideration of what constitutes a valid intervention in text. As Judea Pearl points out in *The Book of Why*, “Causal analysis is emphatically not just about data; in causal analysis we must incorporate some understanding of the process that produces the data” [12].

Early implementations combined Bayes’ rule with computing power to draw causal links between concepts using a priori knowledge. Modern approaches extend this foundation through do-calculus—a mathematical framework for determining when causal effects can be inferred from observational data—and sophisticated graphical models that can represent complex causal relationships [9].

The central challenge in developing these systems lies in what philosophers call *the problem of induction* —how to reliably infer general causal principles from specific instances [13]. Induction represents the process of deriving generalizations from particular cases, while deduction applies these generalizations to new instances. This challenge is particularly acute in language processing, where the high dimensionality of text and the complexity of semantic relationships make it difficult to distinguish genuine causal effects from spurious correlations [4].

A promising framework for addressing this challenge comes from Harriman’s analysis of inductive reasoning in physics [13]. Harriman argues that human causal understanding develops hierarchically—beginning with direct perception of simple causal connections and gradually building to more abstract causal reasoning through the development of appropriate concepts. This framework suggests that attempts to implement causal reasoning in AI systems may be hampered by trying to begin at too high a level of abstraction, without first establishing foundations analogous to humans’ basic causal perception. As we examine the current state of Causal AI in legal reasoning, this developmental perspective provides valuable context for understanding both the challenges and potential solutions.

These fundamental challenges in causal inference for language processing become even more pronounced in legal contexts, where interpretation requires not only understanding statistical patterns but also complex legal reasoning and domain expertise. As we will explore, the application of causal frameworks to legal language processing must address both the general challenges [4] and the specific requirements of legal interpretation discussed in the following sections.

### 1.3. The Lack of Empirical Context in Legal Texts

Legal concepts require particularly acute expertise to be understood. Terms evolve over time and through interpretations that may require extensive knowledge. For example, a single article of a country’s Constitution can generate volumes of analysis when examined word by word.

This illustrates that the immediate context of a legal text is insufficient. Legal formulations come with their history and intent beyond natural language. As a modification of Firth’s famous punchline: You shall *not* know a legal word by the company it keeps [14] (p. 11).

The international element further complicates this, as the rule of law differs between countries. Therefore, NLP techniques for normative natural languages cannot be the same as those for non-normative natural languages.

### 1.4. Relation Between Causality and Legal Language

Traditional AI systems for automatic language processing are correlation machines. Marcos [15] summarizes Marcus (2020b) [16], explaining that Large Language Models (LLMs) tend to rely on superficial statistical patterns rather than the adaptable reasoning characteristic of human cognition. However, correlations can indicate confounding relationships between concepts. This is particularly relevant in legal language processing, where semantic causality helps distinguish misleading correlations from valid ones [17].

A striking example is the COMPAS system used in U.S. courts to assess recidivism risk. While the system showed correlations between demographic factors and recidivism rates, these correlations masked underlying discriminatory biases:

“… blacks are almost twice as likely as whites to be labeled a higher risk but not actually re-offend, [whereas COMPAS] makes the opposite mistake among whites: They are much more likely than blacks to be labeled lower-risk but go on to commit other crimes.” [18]

This exemplifies Simpson’s paradox [19], where a correlation valid from a mathematical perspective becomes discriminatory from a legal standpoint. Causal AI techniques could potentially address this by examining *what if* scenarios to establish genuine causal links rather than relying on correlations.

### 1.5. Legal Assessment in Causal Models for Legal Language Processing

Recent research has explored various approaches to causal linkage in legal texts. Liu et al. [17] propose a Graph-based Causal Inference (GCI) framework that recognizes word groups from key elements in legal texts. Their work, along with others in the field, points to both the potential and challenges of implementing causal analysis in legal language processing.

Similarly, in a study of U.S. states from 1965 to 2012, a novel shift-share design was applied to text-based legislative flows, showing that states enacting more comprehensive and carefully worded statutes experienced higher growth rates [20]. This result was robust to a range of specifications and controls, suggesting that an expanded legislative contract—particularly one embedding contingent clauses—can meaningfully lower uncertainty and encourage business investments requiring relationship-specific assets. The authors’ findings align with the broader hold-up perspective, in which greater legal clarity reduces the risk of opportunistic behavior and thereby stimulates economic activity. Moreover, the text-based natural language processing methods in that analysis, using text as the instrument, provide an illustrative example of how advanced empirical strategies can substantiate the causal role of legislative outputs, echoing the broader need for data-driven causal inference frameworks in legal language contexts.

The integration of classical causal inference principles with legal language processing demands careful consideration of identification strategies and assumptions. The potential outcomes framework highlights fundamental challenges in establishing causation from observational data [21], which become particularly acute when working with legal texts, where controlled experiments are rarely possible. This theoretical foundation underscores the importance of developing Causal AI approaches that can explicitly model counterfactuals while accounting for the complex, context-dependent nature of legal reasoning [7]. Understanding these limitations helps explain both the promise and constraints of current Causal AI applications in legal contexts, setting realistic expectations for what automated systems can achieve.

Since the context of our research leads us to evaluate the possibility of mathematizing legal rules through causal links, while considering the requirements of energy efficiency and computational scalability, this requires mindful reflection on the explainability, algorithmic transparency, and trustworthiness of NLP models [22] while working with reliably extracted legal information [23,24].

### 1.6. Objective

The intersection of Causal AI and legal language processing demands methodological rigor that transcends traditional narrative reviews. While individual studies have illuminated specific aspects of this relationship, the field’s evolution and inherent interdisciplinarity create an epistemic challenge that only systematic investigation can adequately address. A systematic review methodology offers a structured framework for critically examining how causal inference mechanisms interact with legal reasoning’s distinctive characteristics—its narrative functions, its requirement for interpretability, and its fundamental role in maintaining the rule of law.

Through a comprehensive analysis of the literature guided by the Preferred Reporting Items for Systematic Reviews and Meta-Analyses (PRISMA) methodology [25,26], we examine both the technical feasibility of Causal AI in legal contexts and its epistemological foundations as well as its normative implications. This methodological choice enables us to move beyond mere technological assessment toward a deeper understanding of how causal frameworks might preserve—or potentially transform—law’s essential functions while addressing its computational challenges. Moreover, the systematic approach allows us to identify critical gaps in current understanding and potential methodological blindspots that might otherwise remain obscured in more conventional reviews.

Our rigorous examination of existing research provides an essential foundation for future investigations while maintaining careful skepticism about both the promises and limitations of causal approaches in legal language processing.

## 2. Materials and Methods

We used PRISMA’s eight-step process [26] and the PICOC method [27] (p. 41) to frame our research question: What is the current state of research on the challenges, limitations, and potential impact of using Causal AI techniques for automatic legal language processing and its application in legal decision-making compared to non-Causal AI models relying on correlations?

Specifically, our review addresses three questions:

1. What challenges do AI models face in interpreting legal language accurately?
2. How can causal inference improve AI’s understanding of legal language compared to correlation-based methods?
3. How can we address the lack of empirical data in causal analysis of legal texts?

### 2.1. Drafting the Protocol

#### 2.1.1. Inclusion Criteria

**Figure 1:**
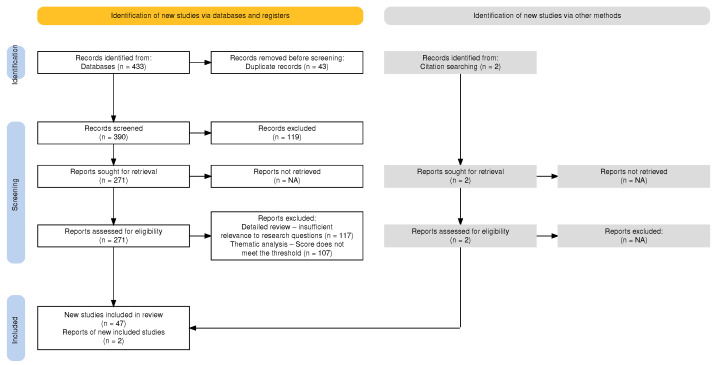
PRISMA flow diagram of the study selection process. Created using the PRISMA Flow Diagram tool [28].

We conducted a literature search across academic databases (Google Scholar, SpringerLink, vLex, Tirant Online), private sector publications (AI Now Institute, The Future Society, OpenAI), and public policy documents (from the European Commission, US Department of Justice, UN, UNESCO, and the Council of Europe). Figure 1 illustrates our systematic selection process. We supplemented database searches with citation tracking to identify relevant papers using different terminology.

We included materials in English, French, and Spanish published between January 2017 and December 2024, focusing on peer-reviewed journals and published theses. Our search employed boolean combinations of terms across three domains: (1) causal analysis terms (e.g., “Causal AI”, “Causal Discovery”, “Causal Inference”); (2) legal applications (e.g., “Legal decision-making”); and (3) NLP terms (e.g., “Legal NLP”).

After removing 43 duplicates from our initial 433 search results, we obtained 390 unique documents for our preliminary analysis.

#### 2.1.2. Exclusion Criteria

We implemented a multi-stage exclusion process: (1) title review excluded 69 documents not directly addressing our research questions; (2) abstract review excluded 50 documents lacking abstracts or that were self-authored; (3) skim-reading excluded 117 additional materials irrelevant to our focus. This process yielded 154 documents that met our preliminary eligibility criteria.

We then scored each article across four dimensions: Causal AI implementation (40 points), legal domain relevance (30 points), NLP applications (20 points), and methodological quality (10 points). Articles scoring 40 points or more (47 articles) formed our final corpus.

#### 2.1.3. Data Extraction

We used a data extraction form adapted from the CASP critical appraisal checklist [29]. We considered declarations of conflicts of interest (financial, ideological, professional, political) and assessed potential publication bias, though neither led to document exclusion.

#### 2.1.4. Data Analysis

We employed thematic analysis principles [30] to analyze our final corpus of 47 articles, beginning with comprehensive reading to identify patterns and categorize findings according to our research questions.

We conducted citation network analysis to identify relevant papers that might have been missed by our keyword-based search. Two thematically relevant cited papers were reviewed against our inclusion criteria.

Our analysis incorporated advanced techniques including LLM-as-a-judge, extraction of practical applications and Causal AI methods, keyword frequency analysis, and cosine similarity checks between abstracts and research questions. We analyzed citation networks to compute disruption measures above 0.5 as defined by [31] and identify key research clusters, providing insight into the evolution and dynamics of this research field. We used various visualization tools including Mermaid diagrams and tables to organize findings, which we synthesized in our Results section.

## 3. Results

The results section of this study aims to provide a detailed examination of the current research on Causal AI techniques in legal language processing, focusing on the main research question: “What is the current state of research on the challenges, limitations, and potential impact of using Causal AI techniques for automatic legal language processing and its application in legal decision-making compared to non-Causal AI models relying on correlations?” This section is structured into four subsections. The first one presents influential trends in the literature and research clusters while showing real-world implementations and potential applications in legal practice encountered in literature. The three subsequent ones correspond to the research questions formulated in the methodology, aiming to build a comprehensive understanding of how Causal AI could reshape legal NLP applications.

### 3.1. Trends Analysis

#### 3.1.1. Disruption Measures

**Figure 2:**
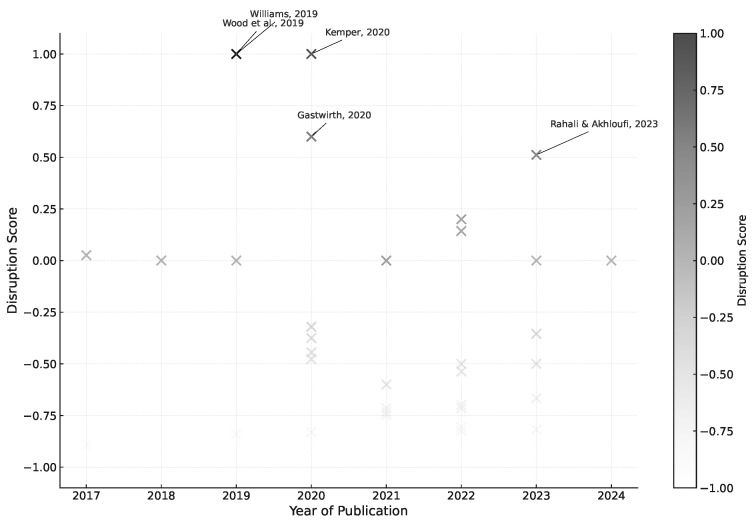
Disruptive articles in the corpus (Disruption Score > 0.5) [32,33,34,35,36]. This scatter plot displays articles with a disruption measure [31] above 0.5, indicating a significant departure from the prior literature. Each point represents an article, positioned according to its year of publication and disruption score. Higher values suggest that subsequent works primarily cite the article itself rather than its references, signaling a shift in research direction. Annotations highlight selected articles with notable disruption scores.

Figure 2 presents the subset of articles in the corpus with a disruption measure [31] exceeding 0.5. These works stand out as potentially influential in reshaping their respective domains, as their citation patterns indicate a break from prior literature. The visual representation contextualizes the temporal distribution of these disruptive articles, allowing for an appreciation of whether such shifts occur steadily or cluster within specific timeframes.

The disruption analysis presented in Figure 2 identifies five papers that significantly departed from previous research trajectories. The introduction of predictive contracting [32] and the framework for ethical AI implementation in legal decision-making [33] represent fundamental shifts in how the field conceptualizes the integration of AI into legal practice. The systematic examination of statistical evidence in civil litigation [34] established new methodological foundations for quantitative legal analysis. However, some papers scoring high on the disruption measure, such as the genomic sequence alignment approach to causality extraction [35] and the analysis of transformer architectures [36], while technically innovative, have had less direct impact on legal applications. This variance between disruption scores and practical influence in the legal domain suggests that methodological innovation alone may not translate into transformative impact in legal AI applications.

The temporal distribution of these disruptive papers reveals an important pattern in the field’s development. Early disruptive works focused on fundamental frameworks for integrating AI into legal practice, while more recent contributions have emphasized technical methodologies. This shift reflects a broader tension in legal AI development between technical sophistication and practical legal application. The high disruption scores of papers focusing purely on technical innovation [35,36] may indicate the field’s increasing emphasis on transformer architectures and sophisticated NLP techniques, potentially at the expense of deeper engagement with legal reasoning challenges.

This pattern informs our subsequent analysis in two crucial ways. First, it suggests that while technical innovations may score highly on quantitative measures of disruption, their impact on legal practice often requires mediation through frameworks that explicitly address law’s unique requirements. Second, it highlights a potential gap in current research: while sophisticated technical approaches proliferate, frameworks for meaningfully integrating these advances into legal practice remain relatively scarce. These insights guide our treatment of various contributions throughout this review, particularly in assessing how technical innovations translate into practical legal applications.

Technical innovation versus practical application directly foreshadows three key challenges that will be explored in subsequent sections: (1) the fundamental difficulty of interpreting legal language with AI models, as evidenced by the high disruption scores of papers focusing on technical methodologies rather than legal understanding; (2) the need for improved causal inference approaches, demonstrated by the emergence of papers bridging technical and legal domains; and (3) the persistent challenge of empirical context in legal texts, reflected in the relatively few highly disruptive papers addressing this specific issue.

#### 3.1.2. Causal AI Applications Trends in Legal Practice

The present analysis revealed several clusters of Causal AI methods emerging in legal NLP research. While the broader field of causal inference encompasses numerous other established methodologies—such as Principal Stratification, Regression Discontinuity Designs, and Time-Series-Based Causal Analysis—Table 1 synthesizes the methods that have been specifically documented in legal NLP applications, organizing each into nine overarching clusters alongside their real-world legal tasks.

Reviewing the citation trends alongside the clusters in Table 1 clarifies why certain methodological innovations (e.g., Causal Representation Learning [37] or Graph-Based Causal Inference [38]) may gain traction in legal NLP. Works that achieve high citation counts often introduce tools or corpora (e.g., large pretraining datasets, specialized benchmarks) that subsequent research employs to tackle causal questions in contract analysis, criminal justice, and policy simulation. In other words, while the table enumerates distinct causal techniques for legal text analysis, their real-world uptake is frequently accelerated when grounded in widely embraced, high-performance language models—facilitating an intersection of advanced representation learning and causal methods. The synergy between large-scale pretrained models [39,40,41] and targeted causal inference practices underscores the growing maturity and sophistication of the field, suggesting that future high-impact publications will likely continue to integrate domain-specific language modeling with rigorous causal frameworks.

**Table 1:** Clustering of causal methods in legal NLP research.

Cluster	Method	Practical Applications	References
1. Causal Reasoning Paradigms	Actual Causation Theory	Automated text analysis; Legal reasoning support systems	[42]
2. Structural & Graphical Approaches	Bayesian Networks	Policy simulation; Legislative impact analysis; Legal argument construction; Judicial reasoning modeling; Societal value change simulation	[8]
	Directed Acyclic Graphs (DAG)	Drug law enforcement	[43]
	Structural Causal Models (SCM)	Policy simulation; Legislative impact analysis; Legal argument construction; Judicial reasoning modeling; Societal value change simulation	[8]
	Structural Equations Model (SEM)	Drug law enforcement	[43]
	Structural Equations	Automated text analysis; Legal reasoning support systems	[42]
	Path Coefficients Analysis	Policy simulation; Legislative impact analysis; Legal argument construction; Judicial reasoning modeling; Societal value change simulation	[8]
	Path-Specific Effects	Drug law enforcement	[43]
	Causal Structural Modeling	Policy research; Legal text analysis	[44]
3. Causal Inference & Identification	Propensity Scoring/Matching	Privacy protection; Criminal justice; Policy research; Legal text analysis	[44,45]
	Inverse Probability Weighting	Privacy protection; Criminal justice	[45]
	Instrumental Variable Regression	Similar charge disambiguation; Automated contract analysis; Bias detection	[37]
	Do-Calculus	Policy simulation; Legislative impact analysis; Legal argument construction; Judicial reasoning modeling; Societal value change simulation	[8]
	Intervention-Based Causal Inference	Policy simulation; Legislative impact analysis; Legal argument construction; Judicial reasoning modeling; Societal value change simulation	[8]
	Causal Inference Network	Electronic court records management; Online legal consulting; Digital case filing; Legal document digitization	[46]
	Graph-Based Causal Inference (GCI)	Automated judicial assistance; Legal advisory services; Criminal case analysis	[38]
	Greedy Fast Causal Inference (GFCI)	Automated judicial assistance; Legal advisory services; Criminal case analysis	[38]
4. Effect Estimation & Assessment	Average Treatment Effect (ATE)	Automated judicial assistance; Legal advisory services; Criminal case analysis	[38]
	Causal Effect Estimation	Contract analysis; Bias detection	[47]
	Long-Term Causal Effect Estimation	Privacy protection; Criminal justice	[45]
	Causal Strength Assessment	Automated judicial assistance; Legal advisory services; Criminal case analysis	[38]
5. Mediation & Indirect Effects	Causal Mediation Analysis	Contract analysis; Bias detection; Privacy protection; Criminal justice; Policy research; Legal text analysis; Similar charge disambiguation; Automated contract analysis	[37,44,45,47]
6. Counterfactual Approaches	Counterfactual Reasoning	Contract analysis; Bias detection; Privacy protection; Criminal justice; Drug law enforcement	[43,45,47,48]
	Attentional & Counterfactual-Based NLG (AC-NLG)	Court’s view generation; Debiasing judgment narratives; Enhanced interpretability of supportive vs. non-supportive cases	[49]
7. Adversarial & Deconfounding	Adversarial Deconfounding	Litigation strategy decisions; Case outcome inference; Legal decision support systems	[50]
	Gradient Reversal Layer	Litigation strategy decisions; Case outcome inference; Legal decision support systems	[50]
8. RL & Decision-Making	Causal Bandits	Civil litigation document review; Anti-corruption audits; Social welfare benefits adjudication	[51]
	Causal Decision-Making Policies	Civil litigation document review; Anti-corruption audits; Social welfare benefits adjudication	[51]
	Constrained Reinforcement Learning	Civil litigation document review; Anti-corruption audits; Social welfare benefits adjudication	[51]
9. Representation Learning (Text/NLP)	Causal Representation Learning	Similar charge disambiguation; Automated contract analysis; Bias detection	[37]
	BERT-Based Embeddings	General use in legal texts analysis	[52]
	Word2vec Adaptation	General use in legal texts analysis	[52]
	Deep Similarity Search	General use in legal texts analysis	[52]
	BERT	Personal injury cases; Patent infringement; Small claims tribunals; Legal document generation; Discovery process	[53]
	GIDBERT	Personal injury cases; Patent infringement; Small claims tribunals; Legal document generation; Discovery process	[53]

The clustering described in the present section offers a nuanced view of how, for example, causality-focused techniques like Instrumental Variable Regression [37] or Adversarial Deconfounding [50] are being used to address particular challenges in contract analysis, bias detection, litigation strategy, and policy simulation. By mapping each Causal AI methodology to specific applications documented in the literature, Table 1 underscores the variety of causal-driven legal tools already in use or under active development, reflecting the field’s growing emphasis on inferring and leveraging causal structures rather than mere correlations.

Trends in these causal methods collectively point to a future where AI can assist legal practitioners in tasks ranging from policy simulation to automated contract review. However, as we detail in the following sections, the trajectory of Causal AI in legal applications is far from straightforward.

- First, we explore the fundamental challenges in interpreting legal language with AI models—challenges that continue to persist despite the sophisticated nature of these emerging causal and correlation-based methods (Section 3.2).
- Next, we examine how causal inference, specifically, addresses these challenges by offering a deeper structural understanding and a more law-aligned approach to reasoning, as opposed to correlation-based methods (Section 3.3).
- Finally, we discuss the scarcity of empirical data and contextual cues in legal domains and show how this gap hampers robust causal analysis (Section 3.4). In doing so, we provide an overview of existing dataset resources and highlight the methodological innovations tackling data limitations.

In short, the trends highlighted here form the backbone of a rapidly evolving landscape, but the subsequent sections underscore the remaining hurdles and the immense potential for future research.

### 3.2. Challenges in Interpreting Legal Language with AI Models

Legal language processing presents formidable challenges for AI models due to the inherent complexity, ambiguity, and context-dependence of legal texts. The specialized terminology, intricate syntactic structures, and nuanced meanings embedded within legal documents demand extensive domain knowledge for accurate analysis. These linguistic intricacies pose substantial obstacles for AI attempting to analyze and make decisions based on legal documents.

#### 3.2.1. Complexity and Ambiguity of Legal Language

As demonstrated by Mamakas et al., even state-of-the-art language models struggle with the length and complexity of legal texts, often truncating important information [54]. Cemri et al. highlighted the need for legal text simplification, underscoring the necessity for sophisticated AI approaches capable of handling the nuances of legal discourse [55]. Hassani et al. draw parallels between legal texts and big data environments, both characterized by vast scale and intricate relationships that complicate the identification of causal links [56]. Barale further emphasizes the difficulty in breaking down human decision-making processes into logical steps and identifying inference and causal links, which are not always apparent in human thought [57].

#### 3.2.2. Limitations of Current AI Techniques

General-purpose NLP techniques often fall short in the legal domain. Lam et al. revealed that semantic similarity between cases does not necessarily translate to legal similarity, highlighting the limitations of standard text analysis metrics in capturing the nuanced legal concepts specific to different cases [58]. This is not a mere detail. It tells us that the metrics that are usually used in computer science for text analysis, for instance to make Retrieval Augmented Generation (RAG), are not suited to the practice of law. In other words, the similarity check between texts has a different meaning in computer science and in law.

This limitation becomes particularly acute when considering causation in legal analysis. According to Pearl, cited by Mik [59], it is impossible for systems to learn causal inference solely from observational data. This presents a critical challenge for legal AI, as understanding causation requires pre-existing world knowledge that cannot be derived purely from pattern recognition. LLMs, confined to learning from text alone, cannot bridge this fundamental gap between correlation and causation that is essential for legal reasoning [59].

As Meneceur & Barbaro emphasize, current AI models face significant challenges due to their reliance on mathematical formalism, which is fundamentally ill-suited for the open, nuanced nature of legal reasoning [60]. This limitation becomes particularly acute when considering law’s fundamental narrative function. Szilagyi states, “law and culture are inseparable; therefore, law and stories are inseparable” [61]. The application of purely correlative methods to legal reasoning risks severing this essential connection between law and cultural narrative, potentially alienating law from the human collective whose movements give its existence meaning.

Current AI approaches in law rely heavily on correlation, pattern matching, and probability distributions derived from historical case data [50,62]. While these methods have achieved some success in tasks like document classification and precedent retrieval, they fundamentally fail to capture the nuanced causal reasoning and narrative understanding essential to legal analysis. Zhang et al.’s analysis of machine reasoning challenges show this limitation, highlighting how traditional neural networks excel at perceptual tasks but struggle with the cognitive reasoning demanded by legal applications [48].

These limitations are exemplified by recent work on transformer architectures like Lawformer [41], which despite sophisticated attention mechanisms and domain-specific training, still fundamentally relies on statistical pattern matching rather than causal understanding. While Lawformer successfully addresses the challenge of processing longer legal documents, it highlights how even state-of-the-art language models remain constrained by their correlational nature.

The limitations of correlation-based methods are also exemplified by research on European Court of Human Rights decisions. While researchers claimed 79% accuracy in categorizing Court decisions, this merely revealed document formatting patterns rather than true legal reasoning, noting that such models would “struggle with a raw account from a future applicant before the Court in Strasbourg” [60] (p. 5).

This challenge is amplified by the increasing complexity and scale of legal data, where identifying genuine causal relationships becomes significantly more complex as data volume and dimensionality increase [56].

Work by Cheng highlights how this poses particular challenges for legal AI systems that must fulfill social responsibilities and ethical principles while maintaining fairness and interpretability [45]. Szilagyi warns that such “machine-ations”, if left unchecked, threaten to destabilize our core institutions and lead to an unintended reordering of our normative framework for the Rule of Law [61].

#### 3.2.3. Need for Domain-Specific Approaches and Causal Reasoning

The necessity for domain-specific AI approaches is evident. Papaloukas et al. demonstrate that legal text classification becomes increasingly difficult as the number of possible classes grows, with performance dropping significantly when moving from broad to specific categories [63]. Their experiments show that domain-specific BERT (Bidirectional Encoder Representations from Transformers; a machine learning model for natural language processing, developed by Google in 2018 [39]. It is designed to understand the context of words in text by looking at the words that come both before and after them)models outperform generic language models and traditional approaches, particularly when classifying detailed legal categories.

A comprehensive survey by Cui et al. identified three primary challenges in legal judgment prediction: inadequate information from facts, the need for complex legal logical reasoning, and the requirement for interpretability [64]. These challenges align with areas where Causal AI could offer significant benefits. Causal models might help in reasoning with incomplete information, capturing complex logical relationships, and providing more interpretable decision-making processes.

This analysis is extended by connecting these challenges to emerging regulatory frameworks like the European General Data Protection Regulation of 2016 (GDPR) and the California Consumer Privacy Act of 2018 (CCPA), which require AI systems to demonstrate trustworthiness, robustness, and transparency—attributes that correlation-based approaches struggle to deliver [48].

The importance of causal reasoning in legal AI is further emphasized by Zhao et al., who highlighted the potential of causal inference approaches to improve AI’s understanding of legal texts [38].

Kemper argues that causal logic in algorithmic legal systems serves verification rather than decision-making [33]. He emphasizes that counterfactual explanations are necessary in the legal field but are primarily found in the explicability of algorithmic decision-making systems evaluating supporting systems.

We will further explore these solutions in Section 3.3 on causal inference.

#### 3.2.4. Explainability and Interpretability

Explainability is a significant challenge in legal AI systems. Kirat et al. proposed a hierarchical framework for algorithmic explanation, highlighting the need for AI models to not only achieve accurate interpretation but also generate contextually appropriate explanations that satisfy diverse stakeholder requirements within specific regulatory boundaries [65]. Rubicondo et al. emphasized the importance of transparency in high-stakes domains [66]. Their findings on AI performance degradation under distributional shifts underscore the potential value of causal approaches in legal AI, where robust and interpretable decision-making is crucial [66].

#### 3.2.5. Ethical and Legal Implications

The narrative and cultural aspects of law present significant challenges for AI systems attempting to interpret and apply legal language [61]. An unduly data-driven approach threatens to strand us in the past, unable to engage in the visioning exercise proposed by law’s narrative arc. Delegating legal decision-making to AI risks transforming the “Rule of Law” into a “Rule by Law”, potentially compromising the integrity of democratic institutions [61].

The COMPAS algorithm case that we mentioned in Section 1.4 provides a stark illustration of these limitations. Despite not explicitly including ethnic origin in its calculations, the algorithm’s correlation-based approach led to biased outcomes through indirect data relationships. Indeed, “cross-referencing of data (including place of residence) indirectly gave too much weighting to this aspect to the detriment of other individual social factors” [60] (p. 8).

Henderson et al. illuminated broader challenges in applying AI to law and public policy. They highlight the “advertisement fallacy”, referring to the naive application of algorithms from one domain to legal and policy contexts without considering their unique challenges [51]. They emphasize the need for AI systems that can provide clear, causal explanations for their interpretations and decisions, crucial in legal contexts where the rationale behind a decision is as important as the decision itself.

The challenges of relying solely on correlational approaches are further highlighted by recent research demonstrating how technical choices in supervised learning can have significant social implications, even when decision rules appear neutral [65]. The analysis reveals that seemingly unbiased algorithmic decisions can generate disproportionate impacts across social groups due to the inability of correlation-based methods to capture underlying causal mechanisms.

#### 3.2.6. Potential Solutions and Future Directions

Addressing these challenges requires a multifaceted approach. Developing more sophisticated language models tailored to legal discourse is essential. The integration of domain-faithful machine learning techniques, as proposed by Balashankar, offers a promising avenue for enhancing the robustness and reliability of AI systems in interpreting and applying legal language [67].

Combining sentence context with event context, as suggested by Khethan et al., could improve causality detection in legal texts [68]. This approach could enhance AI’s ability to capture the subtle implications and context-dependent meanings crucial in legal interpretation. The need for careful adaptation of data mining techniques to the specific demands of legal analysis is underscored [56], suggesting that clustering algorithms must align with legally meaningful categories rather than superficial textual similarities.

Collaboration between AI researchers, legal experts, linguists, and ethicists is vital. Developing robust methodologies to assess the technical feasibility of legally mandated explainability requirements is emphasized in our analyzed literature [65], proposing investigating explainability as a potential mechanism for achieving algorithmic fairness and calling for structured collaboration to ensure technical capabilities align with legal requirements while maintaining interpretability and fairness.

In the following section, we will be exploring advanced methodologies that can capture the nuanced causal reasoning inherent in legal analysis.

### 3.3. How Causal Inference Improves AI’s Understanding of Legal Language Compared to Correlation-Based Methods

After having emphasized the challenges in Section 3.2, the present Section examines how causal inference frameworks can enhance legal AI systems by addressing the limitations of purely correlative approaches. We begin by exploring causal inference as a solution, showing how it better captures the nuanced reasoning essential to legal analysis. The empirical evidence supporting these approaches demonstrates their superior performance across multiple legal tasks. We then delve into the mathematical and theoretical foundations that enable such advances, followed by an examination of key implementation challenges and their solutions. Recent innovations in causal relationship extraction showcase promising new directions, while our final discussion of domain knowledge integration highlights the importance of combining technical capabilities with legal expertise. Through these sections, we demonstrate both the significant potential and current limitations of causal approaches in legal AI.

#### 3.3.1. Causal Inference as a Solution

We begin with Chen et al’s work on logically dependent multi-task learning (MTL) that provides insights into how causal inference can address the limitations we highlighted in Section 3.2 [47]. Their research demonstrates that conventional multi-task learning models make problematic assumptions about conditional independence between tasks, failing to capture the inherent logical dependencies in legal judgement prediction. They advocate for a framework for logically dependent MTL using causal inference: causal multi-task learning (CMTL). Unlike traditional approaches that treat tasks as independent, CMTL mimics human reasoning in teamwork. If you think of each legal task as a team and their interactions as dependencies, CMTL is like a smart coordinator that ensures smooth communication and logical consistency. This way, CMTL explicitly captures how the outcome of one legal task influences another, both directly and indirectly. We will explore the mathematical formalization of CMTL in Section 3.3.3.

It is worth considering Xiao et al.’s work on legal event extraction and event-based knowledge representation, which essentially approaches the problem of mapping textual evidence to underlying causal structures. Recognizing events and the causal relations among them is foundational for legal case analysis [46] (p. 412). These events—often composed of a trigger and its associated arguments—mirror the building blocks that causal inference frameworks employ to model legal scenarios. By integrating insights from large-scale event detection benchmarks like LEVEN (Legal Event benchmark; a Chinese dataset designed for event extraction in legal documents, containing annotated legal events and their relationships [69]), researchers can more effectively identify the causal threads that tie together legal facts, thus bridging the gap between descriptive textual representations and the underlying causal mechanisms that guide judicial reasoning [46]. Such efforts complement the broader endeavor to use causal inference for formalizing legal logic, underlining that legal event knowledge extraction and causal inference techniques are not distinct pursuits but interconnected strategies for deeper understanding of legal language.

So’s comprehensive examination of causality modeling in law demonstrates how formal causal frameworks can transcend the limitations of purely correlative approaches while preserving law’s essential narrative function [8]. This aligns with the observation that law’s normative force adheres in language, meaning, and interpretation [61]. Causal frameworks provide a mechanism for preserving these essential elements of legal reasoning that correlation-based approaches cannot capture.

Consider a case where multiple parties may have contributed to harm. While correlation can highlight associations between actions and outcomes, causal modeling can help determine whether each party’s actions were necessary and/or sufficient causes through counterfactual analysis [8]. From an argumentative standpoint, Pryzant’s framework for analyzing reader responses provides additional tools for understanding how different linguistic presentations of evidence might influence behavior, and thus legal decision-making, helping to disentangle the causal effects of language from confounding contextual factors [70].

Sun et al. demonstrated how this principle can be applied in practice through their Law-Match framework [37]. Legal case matching helps identify how similar two cases are, an important task in legal research. Traditional methods rely heavily on comparing the words and sentences in case documents. However, these methods often miss the legal essence, because they do not consider the relevant laws cited in each case. Law-Match uses a two-step method. First, it separates the information in a legal case into two parts: law-related details (the aspects directly influenced by the cited laws, like key legal points) and other details (the unique facts and circumstances of the case). Then, it combines these parts to make a more informed comparison between cases. This framework achieved superior performance in legal case-matching tasks by explicitly modeling the causal relationship between law articles and case similarities [37].

Overall, research reveals how causal modeling can formalize traditional legal tests like the “but-for” test in tort law [8], while preserving the essential narrative and interpretative functions of law [61]. Through the application of do-calculus and counterfactual reasoning, causal models can represent sophisticated legal concepts such as necessity, sufficiency, and remoteness that correlation-based methods cannot capture. For example, the *reasonable care* concept has different standards across situations [8] which require narrative understanding to interpret properly [61].

Causal inference offers a promising path forward for legal AI. By providing a formal framework for representing legal reasoning about causation, it seems to enable more transparent and accountable decision-making processes. Indeed, the ability to explicitly model interventions and counterfactuals aligns closely with legal practitioners’ actual reasoning processes, potentially leading to AI systems that better support legal analysis and decision-making. Keith’s contributions to measuring and adjusting for confounding variables in text data provide concrete steps toward realizing these benefits in practice [44]. These findings build on the Socially Responsible AI Framework, which emphasizes the need for legal AI systems that fulfill both functional and ethical responsibilities while maintaining interpretability and fairness [45]. It demonstrates that causal learning approaches are essential for developing AI systems that can understand and reason about legal relationships in ways that align with human values and societal expectations.

The analysis of emerging reasoning approaches highlights a crucial transition from traditional symbolic reasoning to hybrid approaches that combine neural networks with explicit reasoning capabilities [48].

#### 3.3.2. Empirical Evidence Supporting Causal Approaches

The previous observations have been empirically validated by recent work on the European Court of Human Rights, where Ichim and Grabmair demonstrated that models explicitly incorporating relationships between legal texts and case facts consistently outperform those relying solely on statistical patterns [71]. Their findings align with Williams’ research on predictive contracting, which demonstrates that purely correlative approaches cannot identify the underlying mechanisms connecting contract terms to outcomes—a crucial capability for effective legal AI systems [32]. The analysis of legal judgment prediction systems further validates this concern, demonstrating that models trained purely on observational data often learn spurious correlations that fail to generalize to novel legal scenarios [64]. As we briefly mentioned in Section 3.2, their findings reveal that inadequate information from facts and complex legal logical reasoning remain fundamental challenges that correlation-based approaches struggle to address effectively.

Wang et al.’s comprehensive analysis of Law School Admission Test (LSAT) reasoning tasks provides empirical validation for hybrid systems that combine pre-trained models with task-specific reasoning modules [72]. Their findings highlight the importance of integrating symbolic knowledge with interpretable reasoning steps, which are crucial for addressing the analytical and logical inference challenges characteristic of complex legal reasoning tasks. For example, the use of explicit symbolic modules to process constraints or logical relations enhances the interpretability and accuracy of these systems, particularly in tasks requiring nuanced legal analysis.

Empirical validation of these benefits is provided in the literature, showing how causal learning approaches significantly outperformed traditional correlation-based methods across multiple datasets and evaluation metrics [37], while others demonstrated even stronger results through the integration of causal inference with neural architectures, achieving significant improvements in prediction accuracy across multiple legal tasks [38]. Together, these studies provide robust empirical evidence supporting the conclusion that hybrid systems, leveraging both symbolic reasoning and neural representations, excel in tackling complex legal reasoning challenges [72].

Successful legal reasoning systems must integrate symbolic and neural components to achieve robust performance [72]. While pre-trained language models offer a strong foundation for understanding legal language, their effectiveness is significantly enhanced by task-specific architectural choices and explicit reasoning modules. These modules enable systems to interpret constraints, deduce logical implications, and address the nuanced analytical reasoning required in legal applications, such as LSAT tasks. However, challenges like data sparsity and the scalability of symbolic methods remain areas for future research and refinement.

The empirical success of frameworks like Law-Match [37], Keith’s probabilistic modeling approaches [44], and GCI [17,38], which have demonstrated significant improvements over correlation-based approaches across multiple legal tasks, provides concrete evidence for the practical value of causal approaches in legal AI applications. Through continued development of hybrid approaches that combine causal inference with advanced NLP techniques, the field moves closer to AI systems that can truly understand and reason about legal causation while maintaining compliance with established legal principles and constraints.

Cohen et al.’s research on deep learning applications in legal contexts provides additional validation for these approaches [53]. Their work with BERT models demonstrates how advanced language models can help overcome the limitations of traditional AI in legal applications. Their findings suggest that deep learning models trained on both legal and negotiation texts can achieve superior performance in understanding legal concepts and relationships, particularly when dealing with unstructured data and implicit causal relationships.

#### 3.3.3. Mathematical and Theoretical Foundations

**Figure 3:**
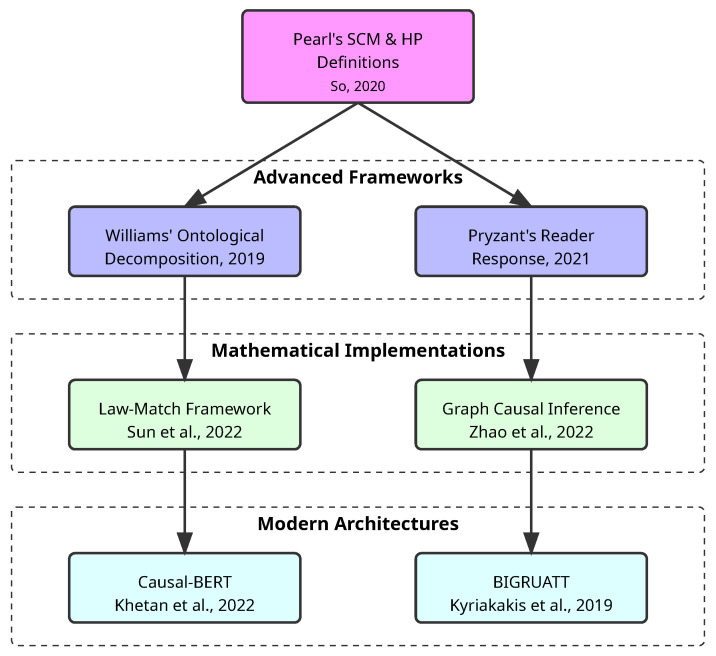
Evolution of causal frameworks in legal AI (2017–2022) [8,32,37,38,68,70,73]. The diagram illustrates the progression from Pearl’s foundational Structural Causal Models (SCM) and Halpern-Pearl (HP) definitions [8] to modern architectures. Color coding represents different developmental stages: foundational theory (pink), advanced frameworks (blue), mathematical implementations (green), and modern architectures (cyan). Arrows indicate direct theoretical or methodological influence between approaches.

The development of causal frameworks in legal AI has followed a clear evolutionary path (Figure 3). Research employs Judea Pearl’s Structural Causal Models (SCM) and the Halpern-Pearl (HP) definitions of actual causality to model legal decisions, providing a mathematical foundation for representing causal relationships in legal reasoning [8]. This approach directly addresses what Pearl terms the “first law of causal inference”—that no causal conclusion can be drawn from purely statistical correlations without additional assumptions about the underlying mechanisms [8].

Other findings complement this theoretical framework by showing how AI systems can decompose legal language into constituent ontological parts to better understand causal mechanisms, rather than just identifying surface-level correlations [32]. Research on reader response algorithms provides additional methodological insights, demonstrating how strategic architectural choices like residualization and adversarial learning can help disentangle confounding factors—a fundamental task in legal contexts where multiple variables may influence outcomes [70]. Building upon these theoretical foundations, the aforementioned Law-Match framework, which operationalizes causal principles by leveraging law articles as instrumental variables to decompose legal case representations into law-related and law-unrelated components [37].

This direction benefits from further advances through a graph-based causal inference (GCI) framework [17] that constructs causal graphs from fact descriptions without extensive manual intervention, enabling more accurate modeling of legal relationships and presenting a method for predicting court decisions by analyzing case details using advanced techniques [38]. The system acts as a decision-support tool for legal professionals. By analyzing the details of a case, it predicts likely legal outcomes in a way that mimics the reasoning process of judges. Its predictions are informed by patterns learned from a large dataset of previous cases, trying to ensure that outputs are both relevant and interpretable within the legal framework.

It combines causal inference, which identifies cause-and-effect relationships in case facts, with a specialized language model, *Lawformer* [41], to process legal texts accurately. By learning from a large database of past judgments, the system predicts applicable laws, charges, and sentences, simulating the combined expertise of multiple legal professionals. This multi-task approach improves accuracy and efficiency, offering lawyers insights into potential outcomes and helping non-experts better understand legal scenarios. The approach balances automation with interpretability, making it a valuable tool for legal decision-making.

The GCI framework constructs a causal graph to model relationships between key factors extracted from case descriptions. The framework processes unstructured legal text as follows:

1. Keyword extraction: Key legal terms, denoted as $\left\{x_{1},x_{2},\ldots ,x_{p}\right\}$, are extracted using the modified YAKE algorithm. This algorithm prioritizes terms based on their frequency, position, and contextual relevance within the text.
2. Clustering: Keywords are grouped into *q* clusters, $\left\{C_{1},C_{2},\ldots ,C_{q}\right\}$, using the *k*-means clustering algorithm. Each cluster represents a set of semantically related legal concepts.
3. Causal graph construction: Clusters are treated as nodes in the graph, and edges are established using the Greedy Fast Causal Inference (GFCI) algorithm. Edges are classified into four types:
  - A→B: A causally influences B.
  - A↔B: A and B share an unobserved confounding factor.
  - A↔B: Ambiguous causation or shared confounding factor.
  - A↔B: Reciprocal causation or ambiguity.
4. Prediction integration: The causal graph informs the prediction model by integrating causal strength metrics, enabling the identification of applicable laws, charges, and sentences.

This framework is formalized mathematically as a directed graph:

(1) $$\begin{array}{c} G=(V,E), \end{array}$$

where $V=\{C_{1},C_{2},\ldots ,C_{q}\}$ are the nodes (clusters of key terms), and E represents the edges with causality types. The causal relationships are weighted by a scoring function S(e), which evaluates the strength and direction of the causal link, ensuring legal consistency in downstream predictions.

This aligns with other findings showing that causally-aware methods can significantly improve the robustness and generalizability of AI systems by capturing underlying data generation processes rather than superficial correlations [45]. Causal-BERT architecture provides a practical mechanism for implementing these theoretical insights, demonstrating how masked event context and pre-training on diverse datasets can help models learn the implicit structural representations needed for robust causal inference [68]. These findings—that models pre-trained on out-of-domain data can perform well on low-resource tasks—are particularly relevant for legal applications, where annotated causal datasets are often scarce.

The mathematical foundations for causal understanding in legal language have been significantly enhanced by both Chen et al.’s introduction of label transfer mechanisms in CMTL [47] and Ritov et al.’s development of conditional parity [43].

On the one hand, the CMTL approach is formalized through a novel mediation assumption that explicitly models the causal relationships between tasks:

(2) $$\begin{array}{c} P\left(Y^{m},Y^{n}|X\right)=P\left(Y^{m}|X\right)P\left(Y^{n}|X,Y^{m}\right), \end{array}$$

where $Y^{m}$ and $Y^{n}$ represent different legal tasks, and X represents the input text [47]. This formalization captures the crucial insight that legal tasks are not conditionally independent, but exhibit causal relationships that must be explicitly modeled. This implementation through label transfer (LT) and Gumbel sampling (GS) provides a practical mechanism for capturing these dependencies while mitigating cascading errors. LT enables the model to reuse label embeddings from lower-level tasks to inform predictions for higher-level tasks. Specifically, the transferred hidden states for task *k*, denoted as ${\mathrm{th}}_{i}^{k}$, are computed as follows:

(3) $$\begin{array}{c} \;{\mathrm{th}}_{i}^{k}={\vec{\mathrm{LSTM}}}^{\left(\mathrm{LT}\right)}\left({\mathrm{le}}_{i}^{k-1},{\mathrm{th}}_{i}^{k-1}\right), \end{array}$$

The arrow on Long Short-Term Memory ($\vec{\mathrm{LSTM}}$) denotes a forward-directional LSTM, which processes sequences from left to right. This LSTM layer takes label embeddings (${\mathrm{le}}_{i}^{k-1}$) from the previous task and the hidden states (${\mathrm{th}}_{i}^{k-1}$) from the previous task’s transfer process as inputs, producing new hidden states (${\mathrm{th}}_{i}^{k}$) that incorporate information from both sources for the label transfer mechanism.

This mechanism allows the model to propagate information across tasks in a logically consistent manner, aligning with the mediation assumption. GS is then used during training to sample from predicted probability distributions, ensuring smoother gradients and avoiding train-test discrepancies by approximating counterfactual reasoning [47].

On the other hand, while traditional fairness metrics often focus on statistical parity, *conditional parity* (CP) frameworks emerge directly from causal reasoning principles [43]. CP extends beyond surface-level statistical relationships by explicitly modeling how protected attributes causally influence outcomes through various pathways. This causal grounding makes CP particularly valuable for legal applications, where establishing genuine discriminatory mechanisms rather than mere correlations is essential. By examining how outcomes vary across protected groups while conditioning on relevant variables, CP enables the identification of genuine causal mechanisms underlying discriminatory effects. Formally, conditional parity is defined as:

(4) $$\begin{array}{c} L\left(x|a=a,z=z\right)=L\left(x|a=a^{\prime },z=z\right)\;\forall a,a^{\prime }\in A, \end{array}$$

where x is the outcome, a the protected attribute, and z the conditioning variable. This ensures outcome distributions are identical across protected groups when conditioned on z.

This formalization allows for rigorous causal investigation of whether disparate impacts arise from legitimate differences in conditioning variables versus discriminatory mechanisms [43]. The framework’s integration with kernel-based conditional independence tests provides robust validation of causal relationships in legal texts, particularly valuable when empirical data is limited.

For example, *equalized odds*, an instance of CP, requires:

(5) $$\begin{array}{c} L\left(\hat{y}|a=a,y=y\right)=L\left(\hat{y}|a=a^{\prime },y=y\right)\;\forall y\in Y, \end{array}$$

where $\hat{y}$ is the predicted outcome, and y the actual outcome.

CP also supports post-processing, ensuring that any function f(x) inherits fairness if x satisfies CP. This framework provides a robust mathematical foundation for fairness.

The authors demonstrate the framework’s utility through practical applications [43], such as addressing disparities in insurance premiums charged to minority neighborhoods, emphasizing the importance of fairness in automated systems (using the conditional cross-covariance operator). Therefore, they address challenges in large-scale causal extraction, as highlighted by Hassanzadeh et al. [52]. The kernel-based approach offers several advantages over traditional correlation-based methods:

1. It captures non-linear relationships commonly found in legal language.
2. It provides a principled test for conditional independence.
3. It scales effectively to high-dimensional feature spaces, typical in legal applications.

This causal interpretation of fairness metrics enhances the broader project of applying causal inference to legal texts and in contexts related to legal situations, such as avoiding de facto discrimination by automated systems. Where traditional correlation-based approaches may identify surface-level disparities, conditional parity frameworks help isolate genuine causal mechanisms of discrimination [43]. This capability proves especially valuable for legal applications where distinguishing correlation from causation is essential for establishing discriminatory impact.

Dymitruk et al.’s research further extends this mathematical foundation through their investigation of the NESS (Necessary Element of a Sufficient Set) approach, which presents a more restricted version of existing counterfactual approaches in law [42]. Their formalization describes a causally relevant condition as a necessary element of a set of conditions jointly sufficient for the outcome, providing a robust framework for distinguishing genuine causation from mere correlation in legal contexts. This approach aligns with Hart and Honoré’s emphasis on the conditionality of necessary and sufficient conditions, offering a precise mechanism for evaluating complex causal chains in legal scenarios. They announce preliminary results in developing a semi-formal framework based on language to model and evaluate degrees of causation in law, validating it in a vaccine injury case [42].

On this subject, Santosh explored deconfounding methods for legal judgment prediction, aiming to identify genuine causal relationships between legal factors [50]. This approach has the potential to reduce AI’s reliance on spurious correlations and improve its ability to reason with legal concepts in a manner more aligned with human experts.

By leveraging these methods, researchers can more accurately test and validate causality in legal data, ensuring precision in downstream tasks.

The evaluation of causal relationships in legal texts requires frameworks that can handle both local and global dependencies [47].

The DocScript model introduces a novel approach to predicting the logical sequence of events in long-form documents, such as contracts or Wikipedia articles, by leveraging advanced NLP techniques [74]. Unlike prior methods that focus on sentence-level event relationships, DocScript addresses the complexities of document-level analysis, where events are often dispersed across multiple paragraphs and require an understanding of both temporal (time-based) and causal (cause-effect) relationships. The system constructs an Event Logic Graph to represent the interactions between events and employs innovative training tasks that enable it to recognize correct event sequences, reorder scrambled events, and identify connections among events within the document. By incorporating optimal transport techniques, implemented through the Sinkhorn algorithm to align event representations, DocScript provides a more nuanced evaluation of causal understanding compared to traditional classification metrics. The alignment process is mathematically defined as:

(6) $$\begin{array}{c} L_{\mathrm{causal}}=\mathrm{Sin}\mathrm{khorn}\left(E,{\hat{E}}^{\prime }\right), \end{array}$$

where E represents the true causal relationships and ${\hat{E}}^{\prime }$ the model’s predictions. This framework provides a more nuanced evaluation of causal understanding than traditional classification metrics, though it still faces limitations in capturing the full complexity of legal reasoning [74].

The DocScript architecture’s evaluation approach aligns with recent advances in transformer-based models, particularly in handling the bidirectional context essential for legal reasoning [36]. This includes edge-based attention mechanisms and optimal transport-based alignments that build upon sequence-to-sequence architectures’ capabilities while addressing their inherent limitations in processing long-form legal documents.

An analysis of Canadian legal cases demonstrates how SCMs can explicitly represent the direction of influence in legal relationships, capturing the crucial distinction between post hoc ergo propter hoc fallacies and legitimate causal chains [8]. This aligns precisely with methods to measure and adjust for confounding variables encoded in text data, thereby offering a framework for more accurate causal estimates in legal contexts [44]. This capability proves particularly valuable in complex cases involving multiple potential causes, intervening acts, and questions of legal responsibility.

Teney et al. propose *gradient supervision*, a technique that uses counterfactual examples to guide neural network training by aligning model gradients with causal mechanisms [75]. Counterfactual examples are pairs of scenarios with minimal differences that lead to different outcomes, to help the AI identify what truly matters. For instance, in a legal context, comparing a case where a contract breach caused harm with one where it did not helps the AI understand the importance of causality. By aligning its decision-making process with these examples, the system learns to generalize better, handle unfamiliar situations, and make fairer, more reliable decisions based on the core principles of a task rather than irrelevant or biased data patterns. This alignment is achieved through a gradient supervision loss function, which ensures the AI’s decision-making boundaries are shaped to follow the logic indicated by these counterfactual examples. By constraining the AI’s decision gradient—the way it changes predictions in response to input changes—this loss function helps the model focus on meaningful causal relationships, leading to more robust and principled generalization. The gradient supervision loss is defined as:

(7) $$\begin{array}{c} L_{\mathrm{GS}}\left(g_{i},{\hat{g}}_{i}\right)=1-\frac{g_{i}\cdot {\hat{g}}_{i}}{∥g_{i}∥\;∥{\hat{g}}_{i}∥} \end{array}$$

where $g_{i}=\nabla_{x}f\left(x_{i}\right)$ is the model’s gradient at input $x_{i}$, and ${\hat{g}}_{i}=x_{j}-x_{i}$ represents the desired gradient direction derived from counterfactual pairs $\left(x_{i},y_{i}\right)$ and $\left(x_{j},y_{j}\right)$ with $y_{i}\neq y_{j}$.

In other words, having two cases that are very similar but lead to opposite outcomes—for example, one where a breach of contract caused harm and another where it did not—the difference between these cases highlights what truly matters for the decision. This loss function ensures the system pays attention to such meaningful changes. It works by comparing how the system’s *reasoning* shifts when moving from one case to another, ensuring its *logic* aligns with the core differences in the examples. In simple terms, it is similar to giving the system feedback on how to connect the dots between similar cases with different results, helping it focus on the principles that matter most [75].

This approach enhances generalization by encouraging models to learn causal rather than correlational patterns, making it particularly relevant for applications requiring counterfactual reasoning, such as legal causation analysis. This complements SCM by offering a practical training objective that encourages models to learn causal rather than merely correlational patterns [8]. The approach is particularly relevant for legal applications where counterfactual reasoning (“but-for” causation) plays a central role.

Finally, it is worthwhile to mention Kyriakakis et al.’s study on transfer learning for causal sentence detection, which offers significant findings on the scalability and effectiveness of different architectural approaches [73]. The study evaluates several architectures, including bidirectional GRU with self-attention (BIGRUATT), ELMO, and BERT, emphasizing the pronounced benefits of transfer learning in small datasets containing hundreds of instances. These findings are especially relevant for specialized legal domains where annotated data is scarce, exploring how AI can detect sentences describing cause-and-effect relationships. They showed that advanced AI techniques, like using pre-trained models (which learn from general data before focusing on specific tasks), work well even with small datasets, a common issue in specialized fields like law. By testing and improving methods, they demonstrated that AI could effectively identify causality in complex texts, making it a valuable tool for legal professionals to analyze cases, find key arguments, and predict outcomes more efficiently.

The research demonstrates that model performance plateaus with just a few thousand training instances, indicating that increasing dataset size alone does not yield further improvements in causal detection. This aligns with observations on the constraints imposed by limited annotation budgets in legal domains [44] and reinforces the emphasis on the importance of efficient learning strategies in low-data scenarios [51].

The mathematical representation provided for the BIGRUATT architecture is as follows [73]:

(8) $$\begin{array}{cc} H^{f} & =\langle h_{1}^{f},\ldots ,h_{n}^{f}\rangle ={\mathrm{GRU}}^{f}\left(e_{1},\ldots ,e_{n}\right), \end{array}$$

(9) $$\begin{array}{cc} H^{b} & =\langle h_{1}^{b},\ldots ,h_{n}^{b}\rangle ={\mathrm{GRU}}^{b}\left(e_{1},\ldots ,e_{n}\right), \end{array}$$

(10) $$\begin{array}{cc} H & =\langle \left[h_{1}^{f};h_{1}^{b}\right],\ldots ,\left[h_{n}^{f};h_{n}^{b}\right]\rangle \end{array}$$

where H represents the bidirectional encoding of the sentence, ^*f*^ and ^*b*^ the forward and backward directions, and [;] denotes concatenation.

A linear attention mechanism computes attention scores for each hidden state:

(11) $$\begin{array}{cc} {\tilde{a}}_{i} & =u_{\mathrm{att}}\cdot h_{i}, \end{array}$$

(12) $$\begin{array}{cc} a_{i} & =\mathrm{softmax}({\tilde{a}}_{i},{\tilde{a}}_{1},\ldots ,{\tilde{a}}_{n}) \end{array}$$

where $u_{\mathrm{att}}$ is the attention weight vector. The sentence embedding s is then computed as a weighted sum of the hidden states:

(13) $$s=\sum_{i=1}^{n}a_{i}h_{i}.$$

Finally, the probability of a sentence being causal is calculated using a logistic regression layer:

(14) $$p=\sigma (u_{p}\cdot s+b_{p}),$$

where σ denotes the sigmoid function, and $u_{p}$ and $b_{p}$ are the parameters of the logistic regression layer.

The BIGRUATT architecture achieved area under the curve (AUC) scores exceeding 0.95 on certain datasets, demonstrating its capability to capture complex linguistic patterns, including implicit causal relationships prevalent in legal texts [73]. Furthermore, their experiments revealed that BERT combined with BIGRUATT consistently outperformed simpler architectures such as BERT+LR in AUC measurements. This suggests the necessity of deeper, task-specific models for robust causal detection in legal applications.

#### 3.3.4. Implementation Challenges and Solutions

The implementation of causal inference in legal AI faces several fundamental challenges. Recent work by [74] demonstrates that extracting causal relationships across long documents requires handling complex dependencies between events that may be paragraphs apart, necessitating sophisticated multi-hop reasoning capabilities. Their findings show that contemporary LLMs struggle with document-level causal reasoning, particularly when temporal sequences span multiple pages, highlighting a key limitation of current approaches.

Causal understanding becomes particularly critical in sequential decision-making (SDM) contexts. Off-the-shelf deployment of existing SDM models to the public sector is insufficient due to the unique challenges posed by legal and policy constraints [51]. SDM algorithms must adapt to handle batched and delayed feedback in legal contexts, where the consequences of decisions may not be immediately observable and may arrive in groups rather than individually [51].

Zhao et al. [38] and Keith [44] extend this capability. In the first case with the aforementioned GCI framework, by automatically extracting and clustering key legal concepts from case descriptions, causal relationships were established between these elements through a combination of sliding window attention mechanisms and global attention patterns [38]. In the second case, by developing methods for identifying causal relationships from text data even with minimal supervision, it was shown how latent disjunction models can aggregate evidence across multiple textual mentions to make entity-level predictions, thus validating these benefits by showing how proper measurement of textual variables combined with causal modeling can significantly improve the robustness of legal predictions, particularly when dealing with distribution shifts common in legal applications [44]. Such legal applications require consideration of multiple objectives simultaneously—for instance, balancing the need to identify causally relevant precedents while ensuring fair and consistent application across different demographic groups [51].

An illustrative approach for addressing severe confounding in legal text generation is the Attentional and Counterfactual based Natural Language Generation (AC-NLG) model, which leverages *backdoor adjustment* to mitigate data imbalance issues [49]. This method involves a claim-aware encoder for highlighting critical aspects of plaintiff claims alongside fact descriptions, coupled with a pair of specialized decoders that generate two distinct views (supportive vs. non-supportive) by counterfactually intervening on the judgment variable. In practice, the system de-biases the data-driven relationship between fact patterns and legal decisions by treating the potential overrepresentation of supportive outcomes as a confounder. By explicitly modeling judgment plausibility and learning separate pathways for supported and non-supported cases, the AC-NLG framework has demonstrated strong performance gains in both accuracy and fairness. The underlying principle relies on intervening on the input representations so that the dominant proportion of supported cases no longer overshadows the minority class, thus yielding a more balanced, confounder-free perspective. This strategy aligns with our broader emphasis on causal inference for improving legal text modeling, especially where label skew or spurious correlations compromise model generalizability and reliability.

However, these challenges are further complicated by the need to handle both explicit and implicit causal relationships in legal texts. Even state-of-the-art language models exhibit significant limitations in zero-shot temporal relation extraction, suggesting fundamental constraints in their causal reasoning capabilities [74]. Indeed, supervised models often outperform larger language models in capturing complex causal dependencies in legal documents [74]. The same applies for Lawformer’s sliding window attention mechanism that, while improving performance on standard NLP tasks, does not address the fundamental limitation of correlation-based approaches in capturing legal reasoning [41].

Causal models can incorporate contextual variables and their relationships explicitly, while correlation-based methods struggle with such context-dependent meaning [8]. The HP definitions’ evolution, particularly in handling pre-emption and overdetermination scenarios, mirrors the legal system’s own development of causation frameworks [8]. The Law-Match framework demonstrates this principle in practice by using law articles as instrumental variables to identify and separate the contextual legal knowledge from case-specific circumstances [37], while the multi-expert FTOPJUDGE mechanism further refines this approach by explicitly modeling the interdependencies between different legal tasks [38]. Such frameworks must also account for distribution shifts in the small data regime typical of legal applications, where the relationships between variables may change over time due to evolving legal interpretations or societal changes [51]. Furthermore, careful measurement of these contextual variables from text data is essential for valid causal inference, and methods have been proposed to handle linguistic complexity while maintaining interpretable relationships between variables [44].

A critical limitation in current approaches is the lack of robust evaluation frameworks for assessing causal understanding in legal AI systems. While traditional metrics like accuracy can measure basic performance, they fail to capture the nuanced understanding required for legal reasoning. The DocScript architecture [74] introduces novel evaluation approaches, including edge-based attention mechanisms and optimal transport-based alignment, providing potential frameworks for assessing causal understanding. However, these metrics still struggle to fully capture the complex interplay between temporal and causal relationships characteristic of legal texts.

Additionally, the construction of accurate causal models requires domain expertise and careful consideration of contextual factors. Different causal models can lead to different legal interpretations, reflecting the inherent complexity of legal reasoning [8]. This complexity is further emphasized by findings regarding the challenges of bridging formal theories with practical legal reasoning [42]. While formal approaches like NESS and actual causation provide valuable theoretical frameworks, their practical application requires careful adaptation to handle the nuanced and context-dependent nature of legal reasoning. The need for semi-formal frameworks that can accommodate both strict and defeasible rules has been emphasized by the literature, allowing for more flexible and realistic modeling of legal arguments [42].

Table 2 summarizes the key implementation challenges identified across the literature in legal AI causal inference.

Recent research has highlighted additional technical constraints in implementing causal inference for legal AI. The DocScript study [74] reveals that learning longer-range dependencies between events remains a key challenge, with performance degrading significantly as document length increases. This limitation is particularly relevant for legal applications, where causal chains may span entire documents and require maintaining coherent reasoning across multiple sections.

These limitations stem partly from fundamental architectural constraints in current language models. Rahali and Akhloufi indicate that Causal Language Models (CLMs), which form the backbone of many legal analysis systems, are inherently limited by their unidirectional nature [36]. While they can process text either left-to-right or right-to-left, they cannot effectively utilize bidirectional context—a crucial capability for understanding complex legal relationships that may span multiple sections of a document [36]. Even advanced transformer architectures like T5 and BART, while offering improvements through sequence-to-sequence processing, still face challenges in maintaining coherent reasoning across lengthy legal texts [36].

The challenges of ensuring accuracy in causal extraction at scale has also been investigated, particularly when dealing with the nuanced and context-dependent nature of legal language [52]. While large-scale text mining can effectively identify potential causal relationships, the precision of these extractions remains a critical challenge, especially in legal applications where accuracy is paramount [52]. Additionally, the integration of normality orderings and blame attribution into causal models remains an active area of development. Several key challenges have been identified in this space [44]:

1. Measurement challenges: Extracting relevant variables from legal text requires handling linguistic complexity, ambiguity, and diversity while maintaining accurate measurement.
2. Legal expertise is required for annotation, making it costly to obtain large labeled datasets.
3. Distribution shifts: Legal interpretations evolve over time, requiring models that are robust to changes in variable relationships.
4. Legal applications demand transparent and interpretable models that can be validated by domain experts.

Cohen et al. expanded on these challenges [53], emphasizing that:

1. Most disputes are resolved through negotiation rather than court decisions, necessitating the incorporation of settlement data for accurate modeling.
2. Legal and negotiation texts vary considerably, making it difficult to structure data properly for machine learning algorithms.
3. A judicial system that is too predictable may be problematic, especially if the status quo is unfair or if decision-making is heavily influenced by extraneous factors.

These theoretical challenges are reflected in practical implementations. For instance, the effectiveness of the Law-Match framework depends critically on the quality and relevance of the law articles used as instrumental variables [37]. Causal-BERT also reveals additional technical challenges specific to language-based causal detection [68]: the need to handle implicit causal relationships that lack explicit linguistic markers, the challenge of maintaining consistent performance across different writing styles and legal domains, and the difficulty of transferring causal knowledge between different areas of law. These findings reinforce the importance of robust pre-training strategies and careful consideration of model architecture when implementing causal inference systems for legal applications.

In sum, the challenges can be classified as follows [8]:

1. Model construction: Building accurate causal models requires significant domain expertise and careful consideration of variables and relationships. The flexibility in model construction means different valid interpretations may exist.
2. Data requirements: Causal inference often requires intervention data that may not be available in legal contexts. While do-calculus can help, some causal relationships may remain difficult to verify empirically.
3. Computational complexity: Causal inference, especially counterfactual analysis, can be computationally intensive for complex legal scenarios with many interacting factors.
4. Integration with existing systems: Most current legal AI systems are built around correlational approaches. Integrating causal reasoning capabilities requires significant architectural changes.

Additionally, by carefully addressing the unique challenges of sequential decision-making in legal contexts—including multi-objective optimization, batched feedback, and distribution shifts—the field moves closer to AI systems that can truly understand and reason about legal causation while maintaining compliance with policy requirements and legal constraints [51].

#### 3.3.5. Innovations in Causal Relationship Extraction

Recent years have witnessed significant advances in causal relationship extraction methods for legal applications, each offering distinct advantages and addressing specific challenges in the field. Table 3 provides a comprehensive comparison of these methods, highlighting their respective strengths, limitations, and optimal use cases.

Building on this comparative framework, we can examine each method in greater detail.

Causal-BERT represents a breakthrough in handling both explicit and implicit causal relationships without relying on traditional pattern-matching rules [68]. This approach demonstrates particular strength in legal applications where causal relationships often require sophisticated contextual understanding.

The development of Causal-BERT represents a significant advance in addressing these challenges, demonstrating that language models can effectively detect both explicit and implicit causal relationships in text without relying on brittle pattern-matching rules or extensive feature engineering [68]. This approach is particularly relevant to legal applications, where causal relationships are often expressed implicitly and require sophisticated contextual understanding to identify.

Recent methodological innovations have further expanded the toolkit for extracting causal relationships from legal texts.

Building on work with evolutionary algorithms for counterfactual sequence generation, recent methodological innovations have further expanded the toolkit for extracting causal relationships from legal texts [76]. Indeed, evolutionary optimization has shown to generate viable counterfactual sequences while preserving essential structural relationships [76]—this is particularly necessary given that, in legal applications, the ordering and dependencies between events carry significant meaning. Evolutionary approaches consistently outperform both random and case-based baselines in generating high-quality counterfactuals, which provides important validation for more sophisticated generative approaches in legal contexts [76].

Arsenyan and Shahnazaryan explore causal relationship extraction in biomedical contexts using methods such as semantic role labeling (SRL) and open-book question answering (QA) [77]. A notable contribution is their two-sided scoring method, which compares the probabilities of directional relationships between entities to infer causality. This approach is particularly relevant for fields like law, where determining causal direction is critical. Their method involves the following formalization:

(15) Givenentitiesaandb,andacontextC:

(16) P(a∣b,C)vsP(b∣a,C),

where the higher probability determines the causal direction. For instance, if P(a∣b,C)>P(b∣a,C), then b→a (i.e., b causes a). This scoring provides a framework for assessing causal relationships by leveraging language models trained with domain-specific data.

The application of this framework could be extended to legal texts, enabling the extraction and evaluation of causal relationships critical for legal reasoning. By adapting this method to legal language models, it could be possible to address nuances in legal discourse and improve the interpretability of causal inferences.

A framework for mining causal knowledge from extensive text corpora has also been proposed, addressing significant challenges in domains like legal applications [52]. The proposed system employs a combination of unsupervised and weakly supervised methods, effectively navigating the diverse representations of causality inherent in legal language. The framework is designed to identify and retrieve causal relationships while providing a quantitative measure of causality through a scoring mechanism.

The system integrates neural embeddings and advanced language models, such as Word2Vec and BERT, to handle the vast variety of causal expressions in natural language. These embeddings enhance the framework’s ability to analyze and retrieve causally related sentences, enabling robust evidence retrieval. Specifically, the causality scoring mechanism calculates the likelihood of a causal relationship between two entities, (X,Y), using multiple methods, including [52]:

- Directional ratio scoring: The score is computed as:
  (17) $$\mathrm{Causality}\;\mathrm{score}=\frac{\mathrm{Hits}(X\to Y)}{\mathrm{Hits}(Y\to X)}$$
  where Hits(X→Y) and Hits(Y→X) represent the frequency of observed directional causal relations.
- Sentence embedding similarity: Using neural sentence embeddings, the framework calculates the average similarity between the input sentence (e.g., “X may cause Y”) and top-*k* causal sentences from the corpus.
- Combined scoring: A hybrid approach that blends directional ratio scoring and embedding-based similarity for enhanced reliability.

Moreover, their framework’s evidence retrieval capability aligns with the legal need for substantiated causal claims. The retrieved evidence includes metadata, such as source documents, to ensure transparency and traceability [52].

In addition, this approach complements methodologies such as Keith’s text measurement framework [44], enabling scalable identification and validation of causal relationships in legal contexts. By integrating domain-specific adaptations, the system underscores the necessity of legal-contextual fine-tuning [77].

This system’s ability to construct *mind maps* of causal relationships also represents a key innovation. Starting from a small set of seed phrases, it generates visualizable graphs of high-scoring cause–effect pairs, significantly reducing the labor involved in manual causal analysis [52].

On the other hand, Wood et al. introduced *OpBerg*, an algorithm that adapts genomic sequence alignment techniques to identify causal relationships in text through optimal alignments of part-of-speech (POS) representations [35].

OpBerg efficiently identifies cause-and-effect sentences in scientific texts by comparing their grammatical structures. Unlike other methods that rely on word lists or extensive training data, OpBerg focuses on patterns in sentence roles (e.g., verbs, nouns). It uses a recursive mathematical approach inspired by DNA sequence alignment, breaking sentences into parts and finding the best matches based on similarity [35]. The process balances accuracy and simplicity by penalizing unnecessary complexity (like too many splits) while maximizing meaningful alignments.

The recursive framework behind OpBerg can be explained intuitively: each sentence can be conceptualized as a chain of linked parts (its grammatical structure). The algorithm evaluates each part against a reference, asking two main questions: *Should this part continue the alignment, or should we start a new one?* and *Does adding this alignment improve the overall score?* Mathematically, this is represented by equations that balance these decisions [35]:

(18) $$X\left(i,j\right)=\max\left\{\begin{array}{cc} X(i-1,j)+Q, & \mathrm{continue}\;\mathrm{alignment}\\ X\left(i-1,j-1\right)+S\left(a_{i},b_{j}\right), & \mathrm{match}\;\mathrm{parts}\\ X(i,j-1)+Q, & \mathrm{start}\;a\;\mathrm{new}\;\mathrm{alignment}\\ 0, & \mathrm{start}\;\mathrm{fresh}\;\mathrm{at}\;\mathrm{this}\;\mathrm{point} \end{array}\right.$$

where:

- X(i,j): The alignment score at position (i,j), which represents the comparison of the *i*-th element of one sequence (or sentence structure) with the *j*-th element of another.
- X(i−1,j): The score if we continue alignment without matching the current element of the second sequence.
- X(i−1,j−1): The score if the *i*-th and *j*-th elements are matched.
- X(i,j−1): The score if a new alignment starts, skipping the current element in the first sequence.
- *Q*: The penalty for introducing a gap (i.e., skipping elements rather than aligning them).
- $S(a_{i},b_{j})$: The score for matching the *i*-th element of one sequence ($a_{i}$) with the *j*-th element of the other sequence ($b_{j}$). Higher scores indicate stronger alignment.
- 0: The decision to start fresh, ignoring previous alignments.

The algorithm recursively evaluates all possibilities, keeping the optimal alignments without requiring pre-specified parameters. This ensures it can efficiently pinpoint meaningful cause-and-effect patterns while avoiding over-complication. For non-mathematicians, think of it as a clever scoring game: the algorithm moves through the structure of a sentence, choosing the path that maximizes coherence while penalizing unnecessary detours.

OpBerg enhances the traditional Alignment with Gap Excision (AGE) algorithm, a method originally designed for genomic sequence alignment, by introducing improvements, such as avoiding the need for pre-specified parameters (e.g., the number of local alignments) or generalizing alignment strategies to allow efficient processing of linguistic data.

Eventhough OpBerg was not developed nor applied in legal contexts, these innovations make it particularly suitable for applications in Legal NLP, where labeled training data is often scarce. By focusing on the POS representations of sentences rather than words, OpBerg reliably identifies patterns that characterize causal relationships.

The algorithm operates with a complexity of $O(n^{2})$ in time, where *n* is the length of the input POS sequence [35]. This is a significant improvement compared to the naive approaches, which can have much higher complexity for similar tasks (e.g., $O(n^{3})$). However, for massive corpora, or datasets with millions of sentences, this complexity can lead to significant delays and is harder to parallelize than linear operations.

A promising architectural direction for addressing the challenges we highlighted in Legal AI emerges from Singer et al.’s Thrill-K framework, which proposes a three-tiered approach to knowledge organization that could significantly enhance causal understanding in legal contexts [78]. Their architecture distinguishes between instantaneous knowledge (for rapid response), standby knowledge (requiring deeper processing), and retrieved external knowledge (accessing additional sources)—a partition that aligns well with the different types of causal reasoning required in legal analysis. This framework complements existing approaches by providing a structured way to integrate different forms of causal knowledge while maintaining computational efficiency.

The Thrill-K architecture’s emphasis on deeply structured knowledge, particularly its incorporation of dynamic models and causal relationships, offers several advantages for legal applications [78]:

1. Enhanced ability to represent complex legal relationships through multiple knowledge dimensions;
2. Improved handling of context and source attribution—crucial for legal reasoning;
3. Better integration of values and priorities, essential for ethical legal decision-making; and
4. More efficient resource allocation through strategic partitioning of knowledge.

#### 3.3.6. Integration of Domain Knowledge and Constraints

AI models often diverge from established domain knowledge when deployed in real-world legal scenarios, leading to spurious correlations and low accuracy in data-sparse or counterfactual scenarios [44,67].

Evidence suggests that AI models often diverge from established domain knowledge when deployed in real-world scenarios, leading to spurious correlations and low accuracy in data-sparse or counterfactual scenarios [67]. Incorporating domain-specific constraints at all stages of the machine learning pipeline is crucial for building robust predictive models that align with legal principles and reasoning. Careful measurement of text data through an empirical pipeline that combines NLP with causal inference has shown to help reduce these spurious correlations [44]. Other research complements these observations, with an emphasis on addressing the challenges of unsupervised symbolic knowledge extraction and enhancing interpretability in legal reasoning systems [72], further highlighting the necessity of hybrid approaches for legal analytics.

It has been shown that deep learning models must incorporate domain-specific constraints to achieve robust performance in legal applications for several reasons [67]:

1. Models trained purely on observational data often learn spurious correlations that fail to generalize to novel legal scenarios;
2. Incorporating domain knowledge through explicit constraints improves model robustness;
3. The effectiveness of constraints depends critically on proper formalization of legal concepts and relationships; and
4. Continuous refinement and validation of constraints is essential as legal interpretations evolve.

Causal inference in legal contexts is further supported by the analysis of model performance across multiple legal tasks [64], which demonstrate that incorporating domain-specific constraints and structure at all stages of the machine learning pipeline is crucial for building robust predictive models that align with legal principles and reasoning.

These insights complement the existing work on causal frameworks, especially work on measurement and causal inference with text data [44], by providing practical mechanisms for incorporating domain expertise into legal AI systems. The combination of careful text measurement, causal modeling, and domain-faithful learning provides a promising path forward for developing AI systems that can truly understand and reason about legal causation while maintaining compliance with established legal principles and constraints.

We will now explore the last aspect of our research question, examining how the lack of empirical context, a precondition for causal inference, is addressed in the existing literature.

### 3.4. Addressing the Lack of Empirical Context for Causal AI in Legal Texts

This section addresses the lack of empirical context for Causal AI in legal texts. We begin by examining foundational challenges and data limitations that hinder causal analysis in legal domains. We then explore methodological advances and implementation strategies that have emerged to address these constraints. After discussing NLP applications and current limitations, we present theoretical frameworks and available dataset resources. The section concludes with future directions and research priorities for improving empirical foundations in legal Causal AI.

#### 3.4.1. Foundational Challenges and Data Limitations

The challenge of empirical data scarcity in legal causal analysis stems from both structural and methodological constraints. Legal data exist in heterogeneous formats spanning textual documents, metadata, and contextual factors, creating significant barriers to systematic causal inference. The fragmentation of data across jurisdictions and non-interoperable systems further compounds these challenges. Indeed, law and public policy pose unique methodological problems, with challenges in multi-objective decision making, batched and delayed feedback, and the requirement for rational, causal decision-making policies that the machine learning community has not yet fully addressed [51].

As discussed in Section 3.2, the narrative nature of law complicates empirical quantification [61]. A comprehensive review of causality analysis in big data demonstrates how the volume and heterogeneity of modern legal datasets pose fundamental challenges for identifying causal relationships [56], revealing that data mining techniques serve as essential tools for managing this complexity, with entity extraction, clustering, association rule mining, and classification emerging as primary approaches for processing large-scale causal information.

The unstructured nature of legal texts poses significant hurdles for AI models. While most judgments follow a structured template, they vary considerably, making it difficult for machine learning algorithms to yield accurate results without proper data structuring [53]. This aligns with findings regarding the limitations of purely correlative approaches in predictive contracting [32]. Transforming unstructured legal data into structured data is a research-intensive endeavor requiring significant computing power and a skilled interdisciplinary team [53].

Data scarcity in legal AI is also a recurrent challenge mentioned in literature. Legal texts are, by nature, high-dimensional and linguistically complex [44]. The distribution of legal concepts and arguments may vary significantly between different areas of law or over time, complicating AI training. Indeed, models struggle particularly with few-shot learning scenarios, especially in specialized areas of law where training data may be limited [63].

#### 3.4.2. Core Methodological Advances

Recent methodological advances demonstrate promising approaches to address these limitations while preserving law’s narrative integrity. Building upon the GCI framework discussed in Section 3.3, recent methodological advances address data limitations by proposing a sliding window attention mechanism to process long-form legal documents efficiently and dilated sliding window attention to capture relationships between distant elements in legal texts [38]. This integration provides two key advantages: it enables the model to learn from both explicit causal relationships documented in legal texts and implicit patterns identified through statistical learning, and by incorporating multiple expert networks focused on different aspects of legal analysis, the framework can effectively leverage limited training data across related legal tasks.

In response to data scarcity, transfer learning and pre-training on out-of-domain data have been explored to address this challenge. While pre-training can improve performance in low-resource causality detection tasks, it may not be a panacea for all challenges [68]. As previously noted in Section 3.3, transfer learning offers solutions to data scarcity; using as a baseline a bidirectional model with self-attention and transfer learning techniques like ELMO and BERT has been shown to significantly improve performance in causal sentence detection [73]. However, for larger datasets (thousands of instances), performance reached a plateau, and transfer learning provided minimal additional benefits. This pattern may well apply to legal language processing tasks, suggesting that while transfer learning can be beneficial for specialized legal tasks with limited data, it may not be a panacea for all challenges in legal AI. This approach aligns with findings regarding the challenges of limited labeled data in specialized domains, demonstrating how carefully designed architectures can help overcome the constraints of small annotation budgets [70]. This is particularly valuable for legal applications where manual annotation is costly but important relationships must be extracted from large document collections.

Gastwirth emphasizes that to produce accurate results, data-driven models must be trained using both legal and non-legal data [34]. He argues that making predictions based solely on legal precedent can yield inaccurate results, as most disputes are resolved via negotiation and hence are unobserved in the data. This aligns with findings regarding the limitations of purely data-driven approaches in legal contexts [53]. While AI can help predict broad outcomes, achieving precise predictions remains challenging due to the inherent flexibility required in legal decision-making. Some level of inconsistency is inevitable and perhaps desirable in judicial processes, as it allows for adaptation to specific circumstances while maintaining sufficient predictability to ensure legal certainty [53]. Thus, effective legal AI systems must incorporate both court decisions and negotiation outcomes to provide accurate predictions and recommendations [53].

Building upon the Law-Match framework discussed in Section 3.3, which leverages law articles as instrumental variables, we note its particular effectiveness in addressing data scarcity [37]. By employing IV regression to decompose legal case embeddings, Law-Match allows for nuanced analysis of legal texts even in scenarios with limited empirical data. This capability is critical for improving Causal AI applications where empirical context is sparse.

Extending the theoretical foundations discussed in Section 3.3, the introduction of conditional parity offers a robust framework for ensuring non-discrimination in AI systems [43]. Particularly, its mathematically rigorous kernel-based conditional independence tests are invaluable for validating causal relationships in legal texts when empirical data is limited. This approach proves essential for managing the complexity of large-scale legal data while upholding statistical rigor.

#### 3.4.3. Implementation Strategies and Practical Applications

Expanding on Williams’ approach discussed in Section 3.3 [32], data collection strategies include structured data collection through integrated contract management systems, transfer learning and few-shot learning techniques for handling limited training data, NLP for extracting structured variables from unstructured legal texts, and standardized frameworks for collecting and analyzing outcome data. While technical constraints around data sufficiency and representativeness remain, these structured approaches to data collection and analysis can help generate the empirical context needed for robust causal analysis of legal texts.

Building on these foundations, Mathis demonstrated sophisticated techniques for extracting structured empirical data from legal texts, achieving an overall quality score of 87.5% for information extraction [79]. His two-step training process combines Conditional Random Fields (CRF) and Flair algorithms, achieving 90% accuracy for legal reference extraction. The demonstrated success in handling different jurisdictional levels and types of litigation suggests these methods could be adaptable across various legal domains. The integration of Mathis’s information extraction techniques with the graph-based causal inference framework presents a particularly promising direction for populating causal graphs with empirical variables.

These practical applications were further advanced by introducing the generative probabilistic modeling approach for prevalence estimation and a latent disjunction model for entity-event measurement [44], as outlined in Section 3.3. Corpus-level evaluation could be efficient for legal causal analysis, as it allows for the assessment of methods at scale, mirroring the vast body of legal texts that often need to be analyzed in practice [44]. The proposed methods are designed to handle linguistic complexity and ambiguity, which are particularly pertinent in legal texts.

#### 3.4.4. Advanced NLP and Deep Learning Applications

As explored in Section 3.3, advanced NLP models like BERT can be fine-tuned for legal applications [53]. This research demonstrates how fine-tuning BERT for legal and negotiation text can create what they term a “Generalized Intelligence in Dispute Resolution” (GIDBERT). This approach addresses the challenge of limited empirical data by learning from massive volumes of legal and negotiation text, including “the entire corpus of European and American case law, academic papers, textbooks, legislation, and even Talmudic law” [53] (p. 6).

#### 3.4.5. Current Limitations and Implementation Challenges

Despite these advances, significant challenges remain. Chalkidis et al. have revealed persistent difficulties in achieving consistent performance across different jurisdictions, languages, and legal areas through their FairLex multilingual benchmark [62]. Their analysis demonstrates that even state-of-the-art fine-tuning techniques cannot reliably guarantee fairness or effectively mitigate group disparities in legal NLP applications.

Specific vulnerabilities in Legal Judgment Prediction (LJP) systems are also highlighted as shallow, distracting surface signals arising from corpus construction, case distribution, and confounding factors [50]. The expert-informed deconfounding method strategically identifies statistically predictive but legally irrelevant information, demonstrating better alignment with expert rationales compared to baseline models. This work emphasizes the vulnerability of LJP models to irrelevant data and the need for more robust approaches that align with expert legal reasoning.

As mentioned in Section 3.4.1, transforming unstructured legal data requires significant resources [53]. This highlights how relying solely on legal precedent data can yield inaccurate results, as most disputes are resolved through negotiation rather than court decisions.

#### 3.4.6. Theoretical Frameworks and Integration Approaches

Extending the theoretical frameworks discussed in Section 3.3, recent developments point toward promising integrative approaches [8]. The application of formal causal models to describe legal decisions provides a crucial link between theoretical frameworks and practical legal reasoning while promoting accountability and minimizing biases [8]. A significant insight is that causal frameworks can promote accountability and minimize biases in legal decision-making, aligning with ongoing efforts in the AI community to develop fair, accountable, and transparent (FAccT) systems [8].

A semi-formal framework has been proposed, employing defeasible logic to bridge theoretical and practical approaches to causation in law, integrating linguistic analysis with evidential reasoning approaches [42]. This integration of multiple analytical techniques could helps address the empirical limitations by leveraging both explicit causal relationships documented in legal texts and implicit patterns identified through linguistic and logical analysis.

Data mining approaches complement these methodological frameworks—for instance, the graph-based causal inference framework can leverage these techniques to more effectively process large volumes of legal documents [56]. As with the Law-Match framework discussed earlier, data mining approaches can enhance scalability when scaling to large legal corpora [37].

#### 3.4.7. Available Dataset Resources

Recent developments in legal document analysis have produced structured datasets that create new opportunities for Causal AI research, while simultaneously exposing methodological challenges in causation detection. Building upon earlier discussions of causal frameworks, emerging datasets specifically address the complexities of long-form legal documents. A comprehensive survey reveals the existence of 43 legal judgment prediction datasets spanning nine different languages, encompassing civil law, common law, and hybrid legal systems [64]. These datasets represent a significant empirical foundation for developing causal models, offering diverse contexts for studying legal reasoning patterns and decision-making processes.

A promising development in this direction is the emergence of LEXTREME, a multi-lingual and multi-task legal benchmark that incorporates 11 datasets covering 24 languages and multiple jurisdictions [80]. LEXTREME provides standardized evaluation protocols for classification and named entity recognition tasks, highlighting how domain-specific benchmarks can illuminate causal relationships in settings where text length, jurisdictional diversity, and specialized legal terminology pose considerable challenges. By offering a curated set of linguistically diverse datasets, LEXTREME could serve as an empirical backbone for assessing whether causal inference techniques generalize across legal systems and languages, thus reducing the field’s heavy reliance on English-centric or single-jurisdiction corpora.

The temporal nature of legal proceedings manifests prominently in contemporary datasets. Collections like CAIL-Long contain longitudinal data enabling the study of causal effects over time, while datasets such as QAjudge provide annotated rationales and intermediate reasoning steps, offering insights into the causal mechanisms underlying judicial decision-making [64]. These resources prove particularly valuable for understanding how temporal dependencies influence legal outcomes, aligning with earlier discussions of the Law-Match framework’s use of law articles as instrumental variables [37]. Expanding this foundation, LegalBench introduces a specialized dataset focusing on legal reasoning causality, distinguishing between statistical and direct-probative evidence in establishing causal links [81]. This dataset addresses a critical gap in the evaluation of causal reasoning capabilities, particularly in discrimination cases where statistical evidence often serves as the primary mechanism for establishing causation.

A significant methodological challenge emerges from the hierarchical nature of legal documentation. Datasets like TOPJUDGE-CJO, TOPJUDGE-PKU, and TOPJUDGE-CAIL capture complex relationships between legal articles, charges, and prison terms [64], requiring sophisticated causal modeling approaches. The presence of these hierarchical relationships necessitates theoretical frameworks that can account for both direct and indirect causal influences, echoing the conditional parity approaches discussed in relation to AI systems fairness [43]. LegalBench’s approach to dataset construction, involving manual extraction and coding of causality-focused passages, demonstrates the intricate nature of identifying causal reasoning patterns in legal texts [81].

The DocSEP corpus introduces specialized datasets derived from legal contracts and Wikipedia articles [74], presenting unique challenges for causal analysis with documents averaging 2479 words for contracts. The significant word distance between related events (averaging 212 words) necessitates models capable of maintaining coherence across extended textual spans. This characteristic aligns with LegalBench’s emphasis on understanding complex causal chains in legal reasoning, where statistical evidence must be evaluated within broader contextual frameworks [81].

Recent work by Mathur et al. demonstrates how emerging datasets enable more sophisticated analysis of legal texts. Their two-step training process combines Conditional Random Fields (CRF) and Flair algorithms, achieving 90% accuracy for legal reference extraction across different jurisdictional levels [74]. This success in handling jurisdictional variations suggests potential approaches for developing generalizable causal frameworks.

The integration of these diverse datasets creates new possibilities for Causal AI research in legal domains. Building upon the GCI framework discussed previously, researchers can now validate causal models across different legal systems and jurisdictions. The multi-task nature of many datasets, combining charge prediction, article recommendation, and prison term determination, provides natural experimental settings for studying causal relationships in legal reasoning. However, the successful extraction of causal relationships requires careful consideration of document-level context and the ability to maintain coherence across extended textual spans [74].

Particularly promising is the presence of datasets with explicit reasoning chains, such as those found in Court-View-Gen and AC-NLG collections [64]. These datasets enable researchers to study not just correlations between case facts and outcomes, but the causal mechanisms through which legal professionals arrive at their decisions. LegalBench extends this capability by providing annotated examples distinguishing between statistical and direct evidence in causal reasoning, offering a valuable resource for developing and evaluating automated approaches to legal causation analysis [81]. This development aligns with the approach on combining legal and non-legal data for accurate predictions [34], while highlighting the challenges of maintaining causal consistency across extended document spans.

## 4. General Discussion and Synthesis

### 4.1. Practical Implementation Guidelines

Causal AI research has increasingly focused on legal applications through structured models and counterfactual approaches. Actual Causation Theory [42] serves as a foundation for automated text analysis and legal reasoning systems, identifying direct causal links to clarify arguments in legal corpora.

Structural approaches including Bayesian Networks and Structural Causal Models [8] support graphical representation of legal cause-effect relationships, helping evaluate downstream impacts of regulations and judicial decisions. Similarly, Directed Acyclic Graphs and Structural Equations Models [43] clarify causal structures in specialized legal contexts.

For causal effect identification, Propensity Scoring and Inverse Probability Weighting [44,45] help control confounding variables in privacy protection and criminal justice analyses. Instrumental Variable Regression [37] proves valuable for estimating causal relationships from observational data with potential unobserved confounding, offering courts and policymakers rigorous methods for inferring legal causality.

Adversarial Deconfounding techniques and the Gradient Reversal Layer [50] concept address bias and spurious correlations in litigation strategy and legal decision support systems. For regulatory applications, Causal Bandits and Constrained Reinforcement Learning [51] improve resource allocation in civil litigation document review and benefits adjudication.

In judicial narrative generation, the AC-NLG model de-biases the court’s view generation using a backdoor-inspired approach to mitigate confounding from imbalanced training data [49]. By modeling support probability as a potential confounder, it improves both rationale generation and judgment accuracy, demonstrating how counterfactual reasoning creates more interpretable legal texts.

Representation Learning approaches, including Causal Representation Learning [37] and BERT-Based Embeddings [52,53], accelerate legal document analysis by capturing nuanced textual features correlated with specific outcomes. These implementations demonstrate that Causal AI provides practical mechanisms for ensuring fairness, transparency, and efficiency across legal practice, while also revealing patterns and challenges that span multiple dimensions of the field.

### 4.2. Synthesis of Findings

Our systematic analysis of Causal AI in legal language processing reveals both significant promise and substantial challenges across multiple dimensions of legal practice. Our findings highlight the interplay between technological capabilities and legal requirements, manifested in three key areas: interpretative challenges, causal reasoning capabilities, and empirical foundations.

**Figure 4:**
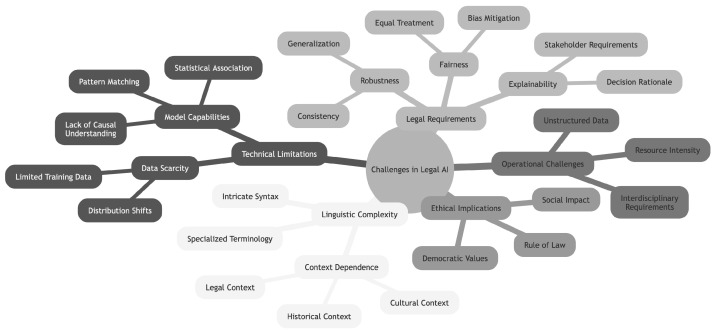
Hierarchical mapping of challenges in legal AI interpretation across five major categories: Linguistic Complexity, Technical Limitations, Legal Requirements, Operational Challenges, and Ethical Implications.

The interpretation of legal language through AI models presents interconnected challenges spanning technical, operational, and ethical dimensions, as illustrated in Figure 4. These challenges emerge from the inherent complexity of legal language, technical limitations of current AI approaches, strict legal requirements, operational constraints, and ethical considerations.

**Figure 5:**
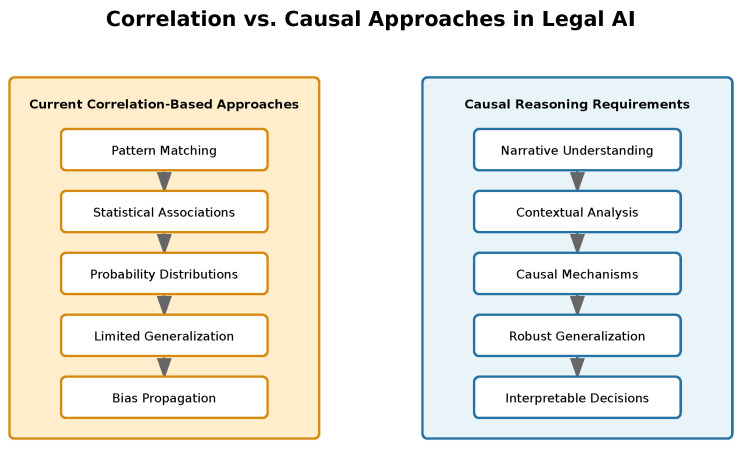
Comparative analysis of correlation-based versus causal reasoning approaches in legal AI, contrasting pattern matching and opaque decision-making with narrative understanding and interpretable decisions.

A fundamental tension exists between current correlation-based approaches and the causal reasoning requirements of legal analysis, as depicted in Figure 5. While correlation-based methods excel at pattern recognition, they fail to capture the nuanced causal reasoning essential to legal analysis [48].

Our investigation reveals how causal modeling can address these limitations by formalizing traditional legal tests while preserving essential narrative and interpretative functions. Through mechanisms like Label Transfer and Gumbel sampling, these models can represent sophisticated legal concepts such as necessity, sufficiency, and remoteness that correlation-based methods cannot capture [47].

The empirical evidence supporting causal approaches demonstrates their superiority across multiple legal tasks. Through frameworks like Law-Match and probabilistic modeling [44], causal methods achieve significant improvements in predictive accuracy while maintaining interpretability. However, these advances face substantial challenges in empirical grounding. The emergence of datasets like LegalBench [81] creates new imperatives for developing architectural solutions that maintain causal coherence across substantial document lengths while preserving legal reasoning’s narrative integrity.

### 4.3. Future Directions and Critical Perspective

Our systematic review reveals both significant promise and substantial challenges in applying Causal AI to legal language processing. While correlation-based approaches demonstrate utility in basic legal tasks, they fundamentally fail to capture the nuanced reasoning essential to legal analysis [61]. Causal frameworks offer a more sophisticated approach, potentially bridging the gap between statistical pattern matching and genuine legal reasoning [8].

Despite promising implementations across diverse areas—policy simulation using Bayesian Networks, automated judicial assistance through GCI, and legal decision support systems leveraging adversarial deconfounding—most represent specialized use cases rather than comprehensive systems deployed at scale [51]. Our analysis revealed two notable patterns: first, many advances in Causal AI techniques that could benefit legal applications originate from other domains; second, current implementations typically adapt general causal approaches to legal contexts rather than developing techniques specifically engineered for legal reasoning’s unique challenges.

Several critical limitations demand attention. First, there is a notable scarcity of real-world case studies demonstrating successful implementations of causal approaches in legal practice. Second, the current literature inadequately addresses how causal models handle legal uncertainty and ambiguity, particularly in scenarios where causation itself is disputed [42]. Third, the computational requirements and scalability challenges of causal approaches remain poorly understood, raising questions about practical deployment in large-scale legal systems [56].

Our analysis of excluded documents revealed a troubling trend: many were excluded because they discussed AI in legal contexts only through transformer architectures, without reflecting on their limitations. This suggests the current fascination with large language models is overshadowing more foundational work on causal reasoning in legal AI, potentially delaying progress on developing robust causal understanding.

The core challenge facing Causal AI in legal domains aligns with Harriman’s developmental pattern of human causal reasoning [13]. Current architectures typically attempt to implement high-level causal reasoning directly through statistical patterns, bypassing the foundational level of causal understanding that Harriman identified as crucial. This helps explain why correlation-based methods continue to dominate despite their known limitations—they sidestep rather than solve the fundamental problem of causal understanding.

Our review also highlights significant gaps in addressing “legal black swan events”—unprecedented cases that defy historical causal patterns [82]. Furthermore, the construction of reliable causal models faces challenges rarely acknowledged: legal datasets often lack clear ground truth for causal relationships, with interpretations varying across jurisdictions and time periods [44]. While the literature extensively critiques bias in correlation-based systems [60], insufficient attention has been paid to potential biases in causal approaches themselves [65].

Nevertheless, recent methodological advances offer promising directions. The emergence of hybrid architectures combining symbolic reasoning with neural representations, exemplified by frameworks like GCI [38] and Law-Match [37], demonstrates potential for systems that can both process legal text efficiently and reason about its implications. The development of sophisticated causal extraction techniques [68] and specialized legal datasets like LegalBench [81] and DocSEP [74] provide essential building blocks for advancement.

Future research directions should prioritize: (1) developing robust evaluation frameworks for assessing causal understanding in legal contexts [74]; (2) creating architectures capable of maintaining causal coherence across lengthy legal documents while remaining computationally feasible [36]; (3) advancing theoretical frameworks that better integrate statistical and direct-probative evidence while preserving law’s narrative functions [61]; and (4) investigating the transferability of these approaches across different legal systems to enhance their generalizability in addressing empirical data scarcity.

A key research priority is bridging the methodological gap between statistical and direct-probative evidence in establishing causation, as highlighted by LegalBench’s annotations. This requires developing models that can distinguish and appropriately weigh different forms of causal evidence while handling substantial document lengths characteristic of legal datasets.

For practitioners, these findings suggest a measured approach to implementing Causal AI. While the technology shows promise for enhancing legal analysis [44], it should be deployed strategically, beginning with well-defined tasks where causal reasoning requirements are clear and consequences of errors are manageable [67]. Success will require close collaboration between legal experts and AI researchers to ensure systems remain aligned with legal principles while leveraging technological advances [45].

The field stands at a crucial juncture where theoretical advances meet practical demands. Future progress depends on balancing technological innovation with preservation of law’s fundamental role in society, while addressing identified limitations—namely, maintaining a realistic perspective on what Causal AI can achieve in legal contexts. Most importantly, it requires recognition that while Causal AI may enhance legal analysis, it cannot replace the fundamental human elements of legal reasoning—the moral judgment, cultural understanding, and narrative interpretation that give law its meaning and legitimacy.

## 5. Conclusions

Our systematic review revealed three concrete findings that directly address our research question about the current state, challenges, limitations, and potential impact of Causal AI in legal language processing:

First, regarding the current state, Causal AI implementations in legal contexts currently exist primarily as specialized solutions rather than comprehensive systems. The most successful current applications focus on narrow tasks: policy simulation using Bayesian Networks [8], which enable explicit modeling of conditional dependencies between legal variables and outcomes, legal case matching through Law-Match [37], which leverages instrumental variables to decompose case representations into law-related and case-specific components, and bias mitigation through adversarial deconfounding [50], which employs gradient reversal layers to identify and remove spurious correlations.

Second, concerning limitations and challenges, our analysis identified three critical constraints in current implementations: (1) the inability to maintain causal coherence across long legal documents, with performance degrading significantly beyond average distances of 212 words between related events [74]; (2) computational scalability issues when processing long documents, which represents the majority of legal texts [81]; and (3) inadequate handling of legal uncertainty, particularly in novel situations or when causation itself is disputed [42]. These limitations suggest that current Causal AI systems, while promising, remain insufficient for general legal reasoning tasks.

Third, regarding potential impact, our findings indicate that Causal AI offers three specific advantages over correlation-based methods: (1) superior performance in legal case matching, with Law-Match demonstrating improvement in accuracy over correlation-based baselines [37]; (2) enhanced interpretability through explicit modeling of legal reasoning steps, as demonstrated by the Graph-based Causal Inference framework [38]; and (3) better handling of bias and fairness concerns through techniques like conditional parity [43]. However, these benefits currently manifest only in narrow, well-defined tasks rather than general legal analysis.

These findings lead to three concrete recommendations for advancing the field: (1) prioritize the development of scalable architectures specifically designed for maintaining causal coherence in long legal documents; (2) focus research on handling legal uncertainty and novel situations through hybrid approaches combining causal inference with symbolic reasoning; and (3) establish standardized evaluation frameworks for assessing causal understanding in legal contexts, particularly focusing on document-level coherence and reasoning transparency.

## Figures and Tables

**Table 2:** Summary of implementation challenges in legal AI causal inference. Challenges are categorized by type and mapped to key literature sources.

Challenge Category	Key Challenges	Sources
Technical & Computational	Long-range dependency handling, Document-level temporal reasoning, Bidirectional context processing limitations, Computational complexity in counterfactuals, Integration with correlational systems, Implicit causal relationship handling, Cross-domain performance consistency	Mathur et al. [74]; Rahali and Akhloufi [36]; So [8]; Khetan et al. [68]
Data-Related	Limited labeled data availability, Small annotation budgets, Settlement data integration, Intervention data scarcity	Keith [44]; Cohen et al. [53]
Implementation	Batched/delayed feedback handling, Distribution shift adaptation, Model interpretability, Unstructured data complexity	Henderson et al. [51]; Cohen et al. [53]
Legal Domain-Specific	Context-dependent meaning, Multiple objective optimization, Predictability-flexibility balance, Legal expertise requirements	So [8]; Henderson et al. [51]

**Table 3:** Comparative analysis of causal extraction methods. Each method presents distinct trade-offs between computational requirements, data needs, and capability scope.

Method	Key Strengths	Limitations	Best Use Case
Causal-BERT [68]	Handles explicit/implicit causality; Strong contextual understanding; Minimal feature engineering	High computational requirements; Large training data needed; Complex model architecture	Complex legal texts with subtle causal relationships
Evolutionary Algorithms [76]	Sophisticated counterfactual generation; Preserves structural relationships; Outperforms baseline methods	Computationally intensive; Requires significant training data; Complex parameter tuning	Generating counterfactual scenarios in legal reasoning
Two-Side Scoring [77]	Clear directional causality assessment; Probabilistic framework; Simple implementation	May oversimplify relationships; Requires domain-specific training; Limited to binary relationships	Determining causal direction in legal arguments
OpBerg [35]	Works with limited training data; Efficient POS-based matching; $O(n^{2})$ complexity	Limited to pattern-based causality; May miss complex relationships; Requires POS accuracy	Quick causal analysis with limited training data
Thrill-K [78]	Structured knowledge organization; Multiple knowledge tiers; Strong domain integration	Complex architecture; Resource intensive; Requires knowledge engineering	Complex legal reasoning with multiple knowledge sources

## Data Availability

No new data were created or analyzed in this study. Data sharing is not applicable to this article.
